# Anwulignan Alleviates Bone Cancer Pain by Modulating the PPARα/CXCR2 Signaling Pathway in the Rat Spinal Cord

**DOI:** 10.1111/cns.70302

**Published:** 2025-03-13

**Authors:** Yueliang Wang, Qingying Liu, Yingying Jiang, Longfei Mao, Mohamed Zoubaa, Jian Wang, Huilian Bu, Minyu Ma, Jingjing Yuan, Jing Cao, Xiaochong Fan

**Affiliations:** ^1^ Department of Pain Medicine The First Affiliated Hospital of Zhengzhou University Zhengzhou Henan China; ^2^ Department of Neuropharmacology, Beijing Neurosurgical Institute Capital Medical University Beijing China; ^3^ College of Basic Medicine and Forensic Medicine Henan University of Science and Technology Luoyang China; ^4^ Department of Human Anatomy, School of Basic Medical Sciences Zhengzhou University Zhengzhou Henan Province China; ^5^ Department of Anesthesiology, Pain and Perioperative Medicine The First Affiliated Hospital of Zhengzhou University Zhengzhou Henan Province China; ^6^ Institute of Neuroscience Zhengzhou University Zhengzhou Henan Province China

**Keywords:** Anwulignan, bone cancer pain, CXCR2, PPARα, spinal dorsal horn, tumor

## Abstract

**Aims:**

Advanced cancer patients frequently endure severe pain from bone metastases, and few effective treatments for bone cancer pain (BCP) exist. Although Anwulignan is known for its antioxidant, anti‐inflammatory, and antitumor properties, its effects on BCP remain unclear. This study aims to explore the analgesic effects and mechanisms of Anwulignan on bone cancer pain.

**Methods:**

Western blotting and immunofluorescence assessed molecular expression and localization. X‐ray, micro‐CT, TRAP, and ALP staining examined bone destruction in rats. MTT, colony formation assays, and in vivo imaging analyzed tumor changes. RNA‐Seq identified differentially expressed genes, validated by ChIP analysis.

**Results:**

Here, we showed that Anwulignan alleviated mechanical, thermal, and cold hypersensitivity and spontaneous pain, prevented bone destruction, and suppressed local tumor growth in rats with BCP. Furthermore, Anwulignan was firmly bound to proliferator‐activated receptor alpha (PPARα), increasing its thermal stability. Intrathecal (i.t.) injection of PPARα siRNA increased pain sensitivity in naive rats, and PPARα siRNA abrogated the analgesic effect of Anwulignan in BCP model rats. Moreover, the PPARα agonist pirinixic acid reduced BCP hypersensitivity and abrogated the upregulation of CXC chemokine receptor 2 (CXCR2). Importantly, PPARα bound to the CXCR2 promoter region, and Anwulignan could reverse the reduced binding of PPARα to CXCR2 caused by BCP.

**Conclusion:**

Taken together, these results indicate that Anwulignan is a potential antitumor and analgesic agent that exerts its effects via upregulation of PPARα expression to inhibit the expression of CXCR2 and could be used for treating BCP.

## Graphical abstract

Anwulignan enhances PPARα expression in the spinal dorsal horn of rats, facilitating its binding to RXR and forming an active PPARα/RXR complex. This complex translocates to the nucleus, inhibits CXCR2 expression, and alleviates bone cancer pain (BCP) while reducing tumor‐induced bone destruction and burden.

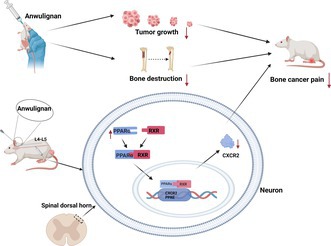

## 1 Introduction

Pain is a prominent symptom experienced by patients with advanced‐stage cancer. Bone cancer pain (BCP) originates either from malignancies that initially develop in the bone or from metastatic bone cancer [1]. Erratic fluctuations and rapid progression characterize BCP; therefore, it is a challenging condition to manage compared with pain caused by inflammatory or traumatic disorders [2]. Among current therapeutic approaches, pharmacotherapy is supported by extensive evidence. The recommended medications primarily include nonsteroidal anti‐inflammatory drugs (NSAIDs), opioids, and antidepressants [3, 4]. However, the efficacy of these treatments varies among individuals [5]. Therefore, novel therapies for the treatment of BCP are urgently needed.

In cancer therapy, natural compounds have recently gained considerable attention. Anwulignan, a derivative of the spice nutmeg, has been recognized for its ability to effectively combat inflammation, oxidative stress, and tumor cell proliferation. Anwulignan protects against renal injury induced by ischemia–reperfusion by suppressing the inflammatory response and regulating apoptotic processes [6]. A subsequent study has revealed that Anwulignan suppresses the growth of non‐small cell lung cancer cells, slows tumor progression [7], and suppresses the metastatic progression of colorectal cancer [8]. Nevertheless, the precise role of Anwulignan in BCP and the underlying mechanism is not fully understood.

The integrity of the skeletal framework is upheld by the delicate equilibrium between bone resorption and formation. Overactivation of osteoclasts or decreased activity of osteoblasts can cause bone diseases such as osteoporosis. Aggressive cancer metastasis to bone tissue frequently disrupts bone homeostatic processes, causing bone erosion and osteolytic lesions [9]. Anwulignan has been shown to promote osteoblast differentiation and exert anabolic effects on bone metabolism in vitro [10]. However, its impact on bone metabolism in vivo has yet to be investigated.

Peroxisome proliferator‐activated receptor alpha (PPARα), a nuclear receptor family member, exerts a vital function in modulating the inflammatory response. PPARα is activated by binding specific ligands, which causes a change in its structure and regulates the transcription of a set of genes. The proteins encoded by these genes are crucial for lipid metabolism and are critical in the inflammatory response [11]. Studies have demonstrated that PPARα agonists can ameliorate mechanical allodynia caused by paclitaxel in rodents [12] and decrease the activation of ipsilateral spinal neurons in a rat model of spinal nerve ligation (SNL) [13]. These findings indicate that PPARα is involved in pain signaling. Moreover, Anwulignan has been demonstrated to increase insulin sensitivity by activating PPARα, suggesting that Anwulignan may exert its effects by acting on PPARα [14]. The chemokine CC motif receptor 2 (CXCR2) is expressed in various cell types and binds to multiple ligands, playing diverse roles in pathophysiology [15, 16, 17, 18]. Recent studies have shown that inhibiting CXCR2 can significantly decrease breast cancer cell‐induced hypersensitivity to touch and the perception of spontaneous pain in mice [19]. Studies have shown that silencing CXCR2 in dorsal root ganglion (DRG) neurons alleviates joint pain, limits the infiltration of neutrophils, and ameliorates walking impairments in a mouse model of gouty arthritis [20]. These findings indicate that CXCR2 is crucial for pain processing. Additionally, studies have shown that PPARα typically influences signaling pathways such as the STAT, NF‐κB, and AP‐1 pathways in a DNA‐binding‐independent manner, thereby suppressing genes associated with the inflammatory response [21]. Concurrently, activation of PPARγ, a family member of the PPARα family, increases CXCR2 expression [22]. Therefore, our theory proposes that Anwulignan can alleviate BCP in rats by influencing the PPARα/CXCR2 signaling axis.

Anwulignan exerts unique effects by modulating the PPARα/CXCR2 signaling pathway in the spinal dorsal horn, reducing the tumor burden and increasing osteoblast activity. This versatile compound has the potential to provide long‐lasting relief from the pain associated with bone cancer.

## 2 Materials and Methods

### 2.1 Animals

Female Sprague–Dawley (SD) rats, with weights ranging from 150 to 180 g, were obtained from the Experimental Animal Center of Zhengzhou University. The protocols governing our animal experiments were formulated in line with the ethical criteria established by the International Association for the Study of Pain. They were reviewed and approved by the Zhengzhou University Animal Ethics Committee. The ethical approval number is 2023‐KY‐1163‐002.

### 2.2 Drugs

The control siRNA (A06001, GenePharm) and a selective PPARα siRNA (A10004, offered by GenePharma with sense and antisense sequences) were obtained. Anwulignan (22,111,603, Pufeide) was dissolved in dimethyl sulfoxide (DMSO). The PPARα agonists pirinixic acid (HY‐16995, MCE) and gabapentin (G154, Sigma–Aldrich) were dissolved in a sterile solution of normal saline (NS).

### 2.3 Cell Culture

The MRMT‐1 rat mammary metastatic tumor cell line was obtained from Crown Bioengineering Co. Ltd., a Shanghai‐based company. The PC12 rat pheochromocytoma cell line was acquired from Procell, situated in Wuhan, China. Before their use, all the cell lines were validated using specific cell‐type identification tests.

### 2.4 Transient Transfection of siRNA

After the PC12 cells and the transfection complex were prepared the PC12 cells were meticulously rinsed using DPBS before the transfection mixture was added. The cells were exposed to the transfection mixture for 6 h at 37°C to facilitate effective siRNA transfection. The cells were harvested for further analysis 2–4 days after the start of the transfection process.

### 2.5 Bone Cancer‐Induced Pain Model

Rats were injected intratibially with MRMT‐1 rat mammary carcinoma cells. The tibia was then exposed, and the proximal medullary canal was accessible by creating an aperture with a 23‐gauge needle. Five microliters of live MRMT‐1 cells (1 × 10^7^ cells) or equal amounts of heat‐inactivated control cells were injected into the medullary cavity using a 10 μL Hamilton syringe. Sterile bone wax was immediately applied to the injection site to prevent any leakage. The incision was meticulously sutured using a layered closure approach to guarantee proper healing [23].

### 2.6 Intrathecal Catheterization and Drug Delivery

Through intrathecal (i.t.) injection, the drug was administered to rats. The procedure for inserting the intrathecal catheter followed the described methods [24]. A polyethylene‐10 catheter (with an outer diameter of 0.61 mm and an inner diameter of 0.28 mm) was inserted into the rats' subarachnoid space via the L5–L6 intervertebral space, with the catheter tip positioned at the L5 spinal segment level.

### 2.7 Behavioral Tests

Before the baseline evaluations, the rats were capable of adapting to the testing environment for a minimum of 3 days. For the assessment of the extent of mechanical pain, von Frey filaments were attached to the underside of the hind paw [25]. Thermal hyperalgesia was evaluated by measuring the time it took for the paw to be withdrawn after exposure to a radiant heat source [26]. The acetone test was employed to evaluate cold allodynia and detect behaviors indicative of heightened sensitivity to cold stimuli [27]. Spontaneous pain responses included the frequency and duration of paw withdrawal and the guarding motions of the resting hind paw on the same side for 10 min [28]. The open field test (OFT) was employed to quantify motor activity within a square arena measuring 100 × 100 × 40 cm^3^ [29]. To ensure impartial outcomes, the observer remained unaware of the treatment conditions throughout the experiment.

### 2.8 X‐Ray Radiography

A radiographic examination of the tibia was performed to detect the degree of bone deterioration [30]. An investigator, unaware of the experimental conditions, conducted quantitative radiographic evaluations.

### 2.9 Micro‐Computed Tomography (Micro‐CT)

The rats were subjected to micro‐CT imaging. After the imaging process, the data were reconstructed with great care using the CTAn software suite, specifically version 1.16.9.0. To thoroughly investigate the three‐dimensional models obtained from the scans, we utilized the CT vox program, version 3.3.

### 2.10 Western Blot Analysis

#### 2.10.1 Total Protein Preparation

The spinal cord was quickly dissected at the lumbar enlargement site. After homogenization, the mixture was incubated on ice for approximately 60 min to ensure complete cell lysis. Following incubation, the samples were subjected to centrifugation at a rapid rate of 12,000 rpm for 15 min at 4°C, thereby enabling the separation of the supernatant from the cellular debris. A BCA protein assay kit was utilized to accurately determine the protein concentration in the supernatant.

#### 2.10.2 Western Blotting

SDS–polyacrylamide gel electrophoresis (SDS–PAGE) was implemented to segregate an equivalent quantity (40 μg) of protein from each sample. The proteins were transferred to polyvinylidene fluoride (PVDF) membranes after separation. To avoid nonspecific binding, the membranes were preincubated for 1 h at room temperature with a TBST‐based blocking solution containing 5% nonfat dry milk. Primary antibodies were administered overnight at 4°C. After the overnight incubation, the membranes were subjected to three rinses with TBST, each lasting for 5 min. This was followed by 1 h of incubation at room temperature with the appropriate HRP‐conjugated secondary antibodies. Immunoreactive bands were subsequently visualized using an enhanced chemiluminescence (ECL) detection system.

### 2.11 Immunofluorescence

Initially, following the appropriate method for extracting the dorsal horn of the spinal cord, sections measuring 25 μm in thickness were prepared. The sections were washed in PBS and then treated with a solution containing 10% goat serum and 0.3% Triton X‐100 to block nonspecific binding. The sections were exposed to these antibodies in a solution containing 1% BSA with 0.3% Triton X‐100 at 4°C for 24 h. Following a comprehensive washing procedure, the sections were then exposed to secondary antibodies that were linked to distinct fluorophores. After being repeatedly rinsed in PBS to eliminate any remaining secondary antibodies, the sections were dried and mounted with anti‐fade mountant to maintain fluorescence.

### 2.12 Cellular Thermal Shift Assay (CETSA)‐western Blotting Assays

CETSA‐Western blotting, which integrates the CETSA with Western blotting [31]. In essence, MRMT‐1 cells were exposed to Anwulignan at a concentration of 60 μM or an equivalent volume of DMSO for 1 h under ambient temperature. The lysates were subjected to heat treatment within 50°C–70°C for 3 min. Next, the lysates were cooled to 4°C for another 3 min. Following thermal cycling, the soluble protein fraction was isolated by centrifugation at 20,000 × g for 20 min at 4°C. The liquid portion with a relatively high concentration of soluble proteins, known as the supernatant, was collected for examination via Western blot analysis.

### 2.13 Molecular Docking Analysis

Anwulignan was obtained from the PubChem database with ID number 10404245 to acquire its 3D structure for docking. Based on the Q07869 template, the 3D structure of P37230 was constructed via a model. Potential active sites were identified using DoGSite. Molecular docking studies were conducted using the AutoDock Vina program, version 1.2.5. Visualization of the three‐dimensional molecular structures was achieved with PyMOL software, and two‐dimensional structural diagrams were generated and presented using Discovery Studio.

### 2.14 RNA Sequencing (RNA‐Seq)

#### 2.14.1 RNA Extraction, Library Preparation, and Sequencing

After 48 h of incubation, PPARα siRNA or control siRNA was used. Each experimental group consisted of three randomly selected PC12 cell samples. The RNA extraction, construction of sequencing libraries, and sequencing procedures were conducted following previously described methodologies [32].

#### 2.14.2 Identification of Differentially Expressed Genes (DEGs) and Gene Ontology (GO) and Kyoto Encyclopedia of Genes and Genomes (KEGG) Analyses

After the initial screening, the clusterProfiler R package was used to conduct a comprehensive GO enrichment analysis on the identified DEGs. Additionally, a statistical assessment was performed to determine the enriched KEGG pathways in these genes. This analysis provides valuable information about the biological functions and pathways that are significantly impacted by PPARα siRNA treatment. The most convincing findings from this extensive investigation were subsequently confirmed via Western blotting.

### 2.15 Chromatin Immunoprecipitation (ChIP)

In our study, the chromatin immunoprecipitation (ChIP) technique was employed to investigate proteinNA interactions within the L4–5 spinal dorsal horn tissue. The DNA fragments generated from the experiment were subjected to immunoprecipitation with 10 μg of anti‐PPARα antibody (Santa Cruz, sc398394) or with IgG as a control, and the specimens were subjected to the antibody and underwent incubation overnight at 4°C. A 10% portion of the sample, known as the input, was reserved for the immunoprecipitation process. The immunoprecipitated protein‐DNA complexes were then extracted, cleaned, and processed for amplification targeting the promoter regions of the CXCR2 gene. The potential binding sites for PPARα within the promoter region of the CXCR2 gene were identified in advance via the JASPAR database as a reference [24]. A comprehensive list of all primers utilized throughout the study is presented in Table 1.

**TABLE 1:** Primers for the CXCR2 promoter region.

No		Sequences (5′‐3′)	Pcs
1	Forward	CGGTCCCCAGCTCAAAATAAAT	22
	Reverse	TGTTTTCTTTTTGCAGTGGATTATG	25
2	Forward	CAAGCTGAGAGGAGGTTAGGTT	22
	Reverse	GCTAGGTTTCCCTGCTATCGAA	22
3	Forward	GACTCAGCCCTGCCTGC	17
	Reverse	ATCAAGGAAACCAGACCCAAGA	22
4	Forward	ACCACTTTACAAGGTCCAGGTG	22
	Reverse	ATAAAAACGGTGTTGCCCTACG	22
5	Forward	CCAAATCAGGTGTTTCTACGGTC	23
	Reverse	ATTATGCCCCATTGTGTGGAAC	22
6	Forward	AATCTATGCTATGTCGGTGGCAGA	24
	Reverse	AGCAGATTGGAGAGAGACAAGGAA	24
7	Forward	GGAGTGCCTTAGAGTAGGGTGT	22
	Reverse	CTTCCTTTCCCACTCCGTGAAG	22
8	Forward	TCTCCAATCTGCTTGCCTTTAGATA	25
	Reverse	CTCCCTCTGTCTCAGAACCCTT	22
9	Forward	AGGGGAGGAGGAACAGAGTAAC	22
	Reverse	TTGGCACTTTCGTTTCTTGTGAG	23
10	Forward	AGAGACCGTTGTGGCAGAAG	20
	Reverse	CCTGGTGATTCCGAGGTGAG	20
11	Forward	GGCATAGGCTGGGAAGGAAG	20
	Reverse	TCTACGATGCTGGGAGTGGA	20

### 2.16 Statistical Analysis

All values are presented as mean ± SEM and analyzed using GraphPad Prism 8.0. Prior to conducting the analysis, all data were initially examined for normality. The Shapiro–Wilk test was employed to evaluate the normal distribution of the data. Provided that the data adhere to a normal distribution with homogeneous variance, Student's *t*‐test was employed for comparisons between two groups, one‐way analysis of variance (ANOVA) for three or more groups, and two‐way repeated measures ANOVA for comparing two variables across two or more groups. Alternatively, Mann–Whitney U and Kruskal–Wallis tests were conducted in cases where the data did not adhere to a normal distribution. A *p*‐value less than 0.05 was regarded as statistically significant.

## 3 Results

### 3.1 Intratibial Injection of Tumor Cells Leads to Bone Destruction and Heightened Nociceptive Sensitivity

**FIGURE 1:**
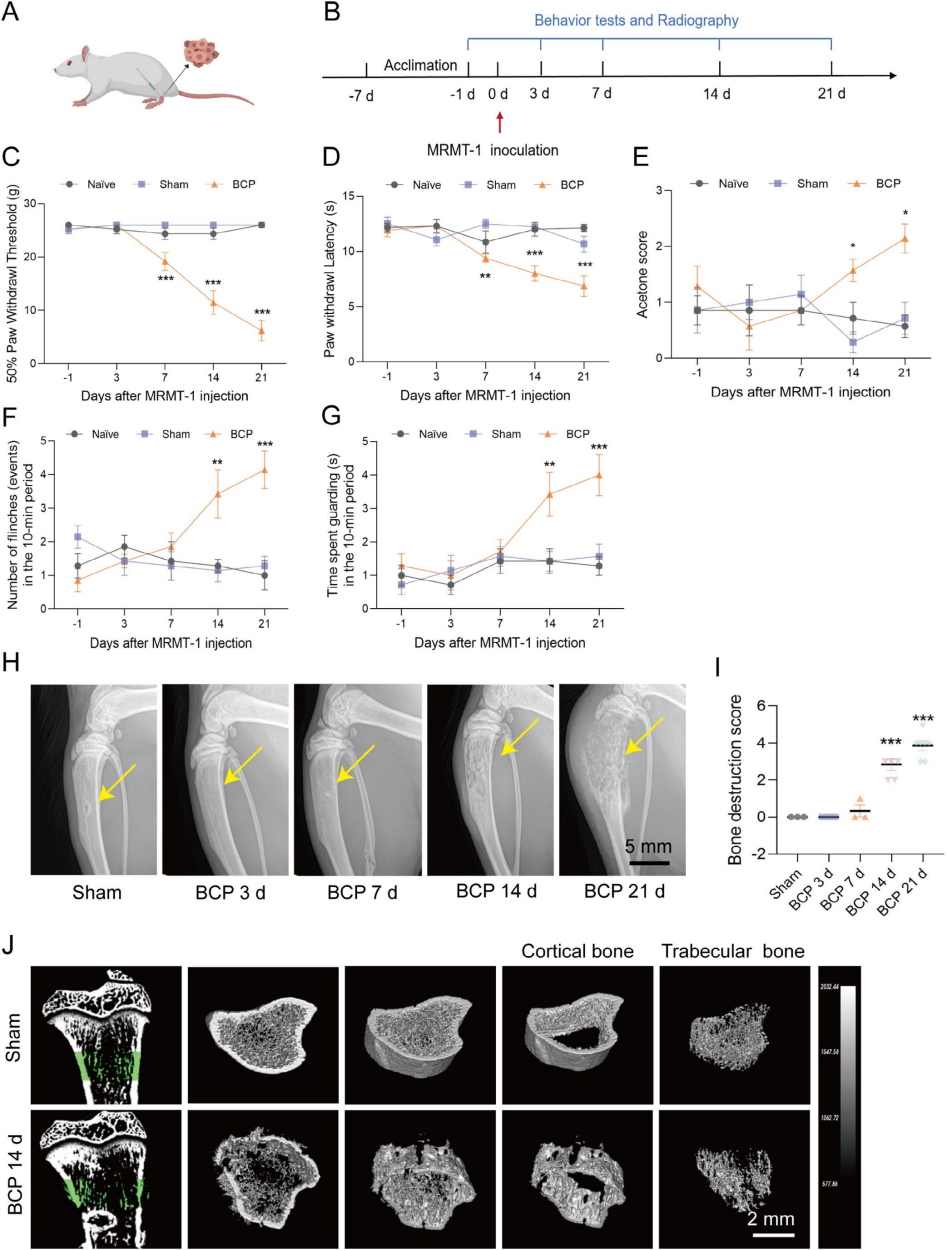
Development of the MRMT‐1‐induced BCP rat model. (A) A rat was injected with MRMT‐1 cells to establish the BCP model. (B) The experimental schedule is depicted in graphs outlining the timing of the behavioral and radiological assessments in the BCP model. (C–G) Animals were categorized into the control (naive), surgical control (sham), and MRMT‐1‐induced BCP groups. Behavioral assessments of the BCP model rats included the 50% paw withdrawal threshold (PWT) (C), paw withdrawal latency (PWL) (D), acetone test score (E), and spontaneous pain behaviors such as flinching (F) and guarding (G), which were measured on Days −1, 3, 7, 14, and 21 post‐treatment. Statistical significance (*p < 0.05, **p < 0.01, ***p < 0.001) in comparison with the sham group was ascertained through a two‐way ANOVA. (H) Radiographic images of the tibia of the affected limb were captured at multiple time points after mock surgery and MRMT‐1 cell injection, revealing the progression of osteolytic destruction associated with cancer advancement, with notable changes observed on Day 21 after injection. (I) Quantitative analysis of the radiographic data. A one‐way ANOVA was employed to establish statistical significance (***p < 0.001) in comparison with the sham group. *n* = 3–7. (J) Micro‐CT images of rat tibiae on Day 14 after MRMT‐1 inoculation and from the sham group revealed various findings: 1. Coronal view of the affected tibia; 2 and 3. Cross‐sectional view of the affected tibia; 4. The Trabecular bone of the affected tibia; 5. cortical bone of the affected tibia. By Day 14 post‐tumor cell injection, the trabecular and cortical bone lesions were evident.

The development of MRMT‐1 tumors led to the destruction of rat bone (Figure 1A). To assess the effects of tumor cell injection on sensory processing, various tests were used to measure different aspects of pain (Figure 1B). In the rats injected with tumor cells, the 50% PWT (Figure 1C) and PWL (Figure 1D) were significantly reduced from Day 7 after surgery and continued until Day 21. Starting on Day 14 after surgery, the acetone test scores of the model group (Figure 1E) presented significant divergence from those of the sham group, and the significant differences persisted until Day 21. Furthermore, spontaneous pain behaviors (including flinching and guarding) (Figure 1F,G) increased significantly from Day 14 after surgery. The behavioral measures exhibited no substantial disparity between the untreated and sham groups.

In addition to the behavioral assessments, imaging methods were used to confirm the successful development of the BCP model (Figure 1H). X‐ray images revealed a pattern of progressively exacerbated bone destruction associated with the progression of MRMT‐1 tumors. On Day 14 post‐implantation, local bone damage was detected, whereas on Day 21, osteolytic lesions were evident (Figure 1I). This approach aligns with the methods established by Honore et al. [33]. Furthermore, the bone density of the affected tibia 14 days after surgery was evaluated via micro‐CT imaging, reconstructed, and compared to that in the sham tibia. On Day 14 after surgery, significant degeneration of the trabecular and cortical bone around the tibia was observed in comparison to the sham group (Figure 1J).

### 3.2 Anti‐Nociceptive Effects of Anwulignan on Rats With BCP

**FIGURE 2:**
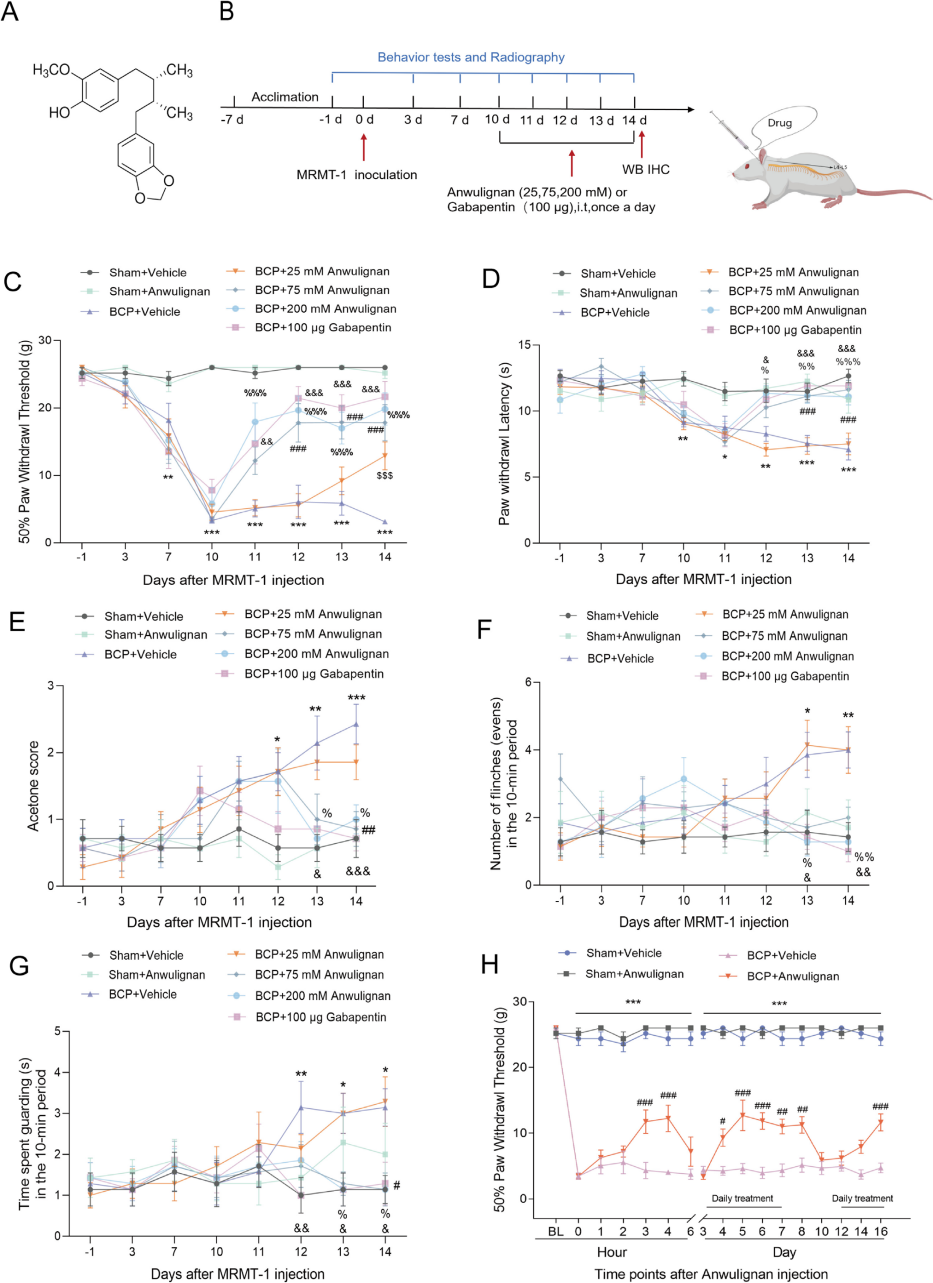
Anwulignan attenuates BCP. (A) The chemical formula of the Sigma–Aldrich Anwulignan formulation. (B) Schematic diagram of the experimental protocols. (C–G) Anwulignan was administered at concentrations of 25, 75, and 200 mM, whereas gabapentin, at a dose of 100 μg, served as a standard treatment and was delivered intrathecally (i.t.) for five consecutive days. Behavioral assessments were conducted at baseline before MRMT‐1 cell injection and subsequently on Days 3, 7, and 10–14 days postinjection. These evaluations included measuring the 50% PWT (C), assessing the thermal PWL (D), conducting an acetone test to gauge cold sensitivity (E), and observing spontaneous pain behaviors such as flinching (F) and guarding (G). *p < 0.05, **p < 0.01, and ***p < 0.001 vs. the Sham + Vehicle group; ^%^ *p* < 0.05, ^%%^ *p* < 0.01, ^%%%^ *p* < 0.001, ^#^ *p* < 0.05, ^##^ *p* < 0.01, ^###^ *p* < 0.001, ^&^ *p* < 0.05, ^&&^ *p* < 0.01, ^&&&^ *p* < 0.001 vs. the BCP + Vehicle group; two‐way ANOVA. *n* = 7. (H) The 50% PWT to mechanical stimuli was evaluated 10 days after the inoculation of MRMT‐1 cells. Compared with vehicles, Anwulignan increased the 50% PWT. *p < 0.05, **p < 0.01, and ***p < 0.001 vs. the Sham + Vehicle group; ^#^ *p* < 0.05, ^##^ *p* < 0.01, and ^###^ *p* < 0.001 vs. the BCP + Vehicle group; two‐way ANOVA. *n* = 7.

Our primary objective was to assess the therapeutic potential of Anwulignan (Figure 2A) in a rat model of metastatic bone cancer (Figure 2B). The findings suggest that the BCP group displayed significantly heightened pain sensitivity compared to the sham group(Figure 2C–G). From Day 10, the pain sensitivity of the rats that received different doses of Anwulignan or gabapentin (100 μg) was significantly reduced, except for the rats that received the lowest concentration of Anwulignan (25 mM), suggesting that Anwulignan provided pain relief in the numerous experiments conducted in this study. Furthermore, Figure 2H shows the effects of various administration methods on mechanical hyperalgesia in BCP model rats. We discovered that BCP significantly decreased the 50% PWT for mechanical stimulation, and this sensitivity remained invariant throughout the study duration in comparison to that of the Sham + vehicle. Notably, the 50% PWT of rats treated with Anwulignan increased within 3 h of the daily application. However, mechanical hyperalgesia returned after the daily treatment regimen stopped. This finding suggests that BCP model rats did not develop tolerance to Anwulignan. (Administration of Anwulignan exhibited no statistically significant influence on locomotor function in BCP model rats (Appendix S1 or Figure S1)).

### 3.3 Biophysical Binding of Anwulignan With PPARα and PPARα Is Localized in Spinal Cord Neurons

**FIGURE 3:**
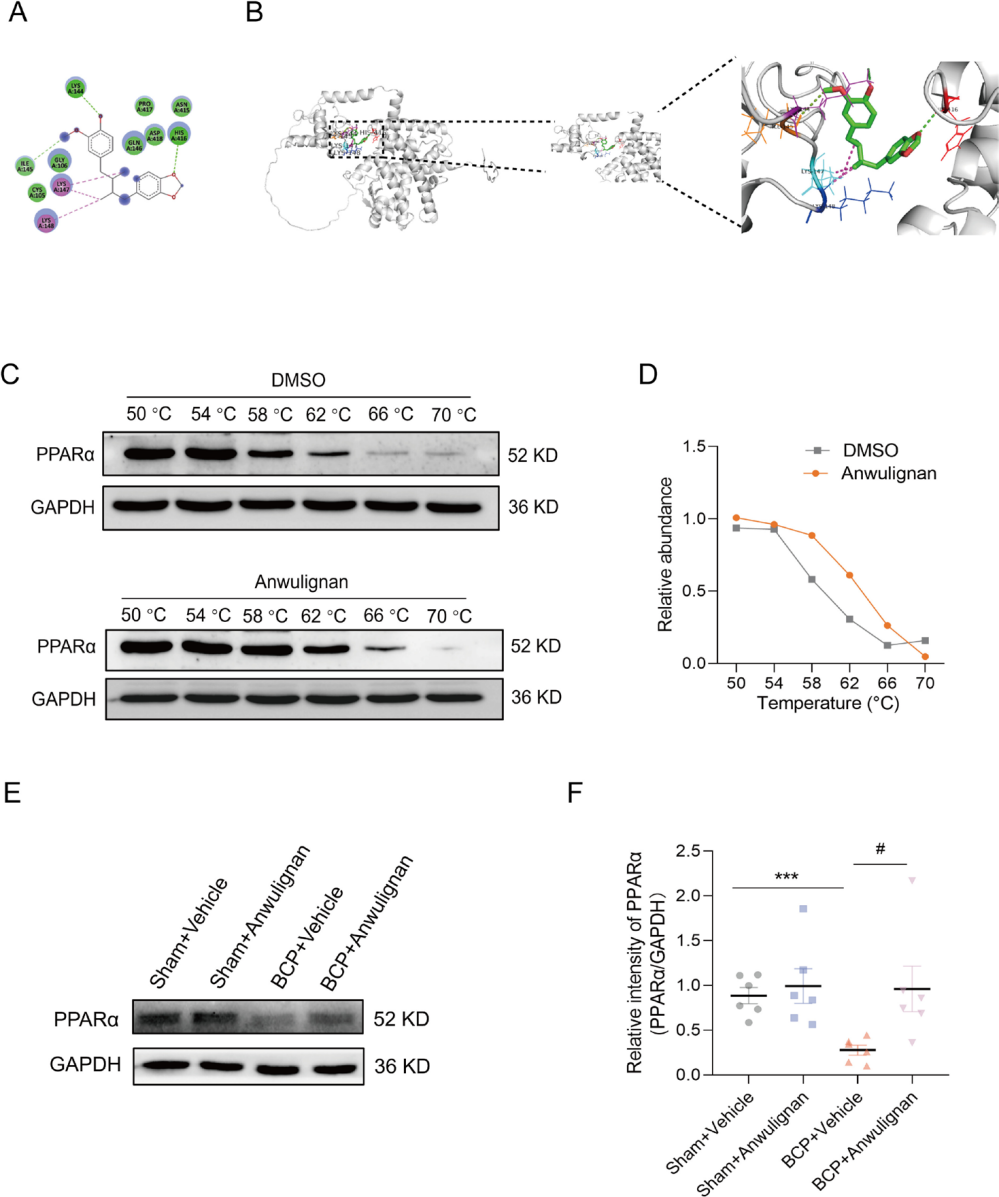
Molecular binding of Anwulignan with PPARα. (A) The chemical composition of Anwulignan is displayed. (B) Predictions of the molecular docking between Anwulignan and the PPARα protein. (C) A CETSA was conducted to evaluate the binding of PPARα to Anwulignan. (D) The thermal stability of PPARα is quantified in the panel shown in (C), indicating an increase due to Anwulignan binding, with a change in the melting temperature (ΔTm) of approximately 3.6°C. (E) Western blot images demonstrated that Anwulignan significantly elevated PPARα expression compared to the BCP group. (F) Statistical analysis of the results from (E) is provided, and six samples were analyzed. An unpaired *t*‐test was used to determine that a significant difference was indicated by ***p < 0.001 compared with the Sham + Vehicle group and by ^#^ *p* < 0.05 compared with the BCP + Vehicle group.

**FIGURE 4:**
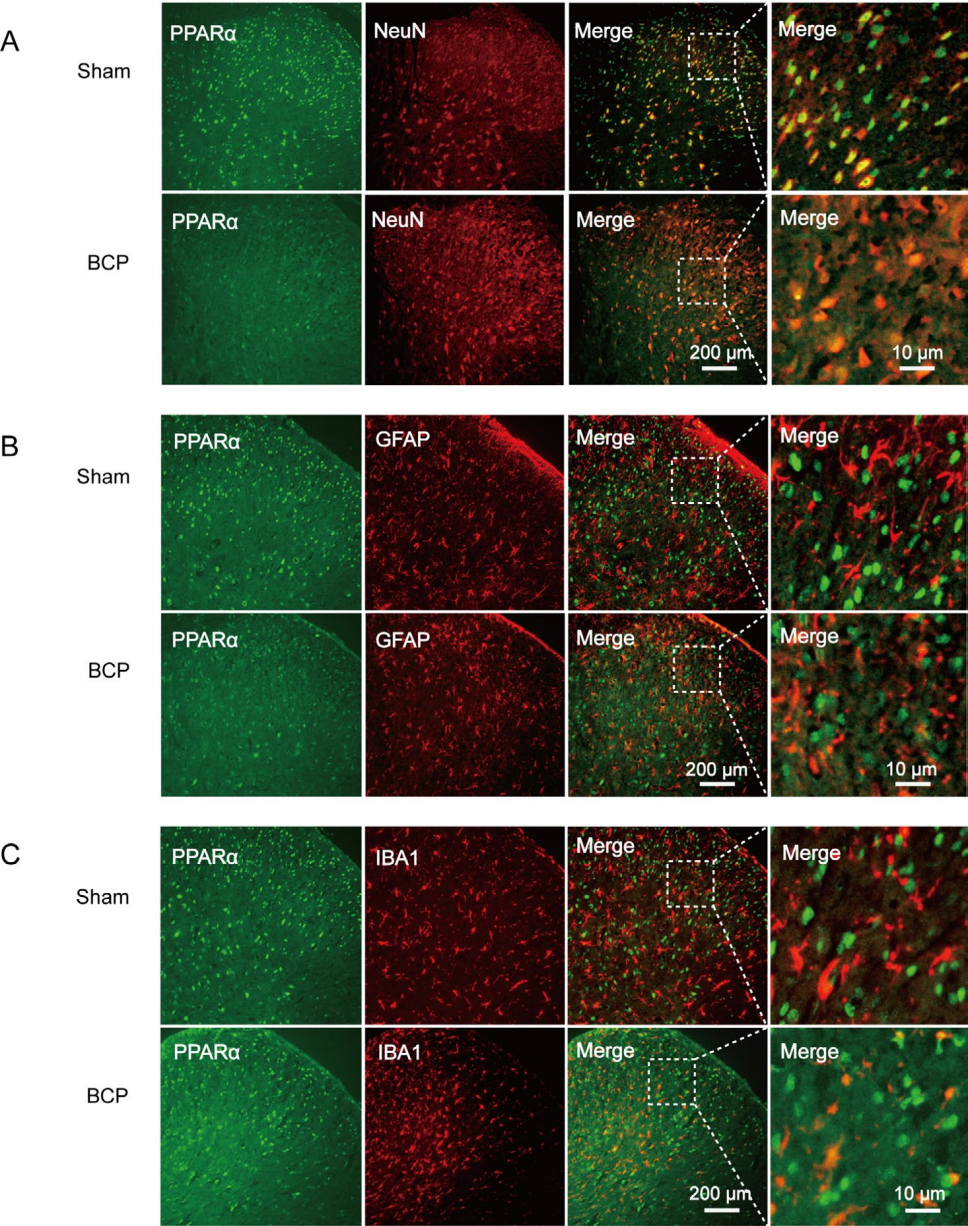
PPARα expression in the spinal cord was reduced following the onset of BCP. The immunohistochemical staining results depicted in (A–C) reveal that PPAR‐α was primarily localized in neuronal cells (A) within the dorsal horn region of rats with BCP, with no detectable expression observed in astrocytes (B) or microglia (C).

Molecular docking studies revealed that Anwulignan (Figure 3A) had a strong affinity for PPARα, with a docking score of −5.648. Anwulignan was discovered to form hydrogen bonds with the LYS144, ILE145, and HIS416 residues of PPARα. The protein forms hydrogen bonds with corresponding bond lengths of 2.08977, 3.54821, and 5.37155 Å (Figure 3B). A CETSA confirmed the molecular interaction between PPARα and Anwulignan. The rationale of CETSA is that the binding of ligands can induce changes in the thermal stability of the studied protein. The binding of Anwulignan was found to increase the thermal stability of PPARα (Figure 3C) by approximately 3.6°C (Figure 3D), suggesting that Anwulignan directly interacts with PPARα in the cellular environment. Furthermore, Western blot analysis of the spinal dorsal horns of the rats given vehicle or Anwulignan revealed that PPARα expression was upregulated in the Anwulignan group compared to the BCP group(Figure 3E,F). These comprehensive molecular binding properties suggest that PPARα is a direct molecular target of Anwulignan. Meanwhile, immunofluorescence results showed that PPARα was localized in spinal cord neurons (Figure 4).

### 3.4 Pirinixic Acid Can Effectively Alleviate Pain in BCP Model Mice

**FIGURE 5:**
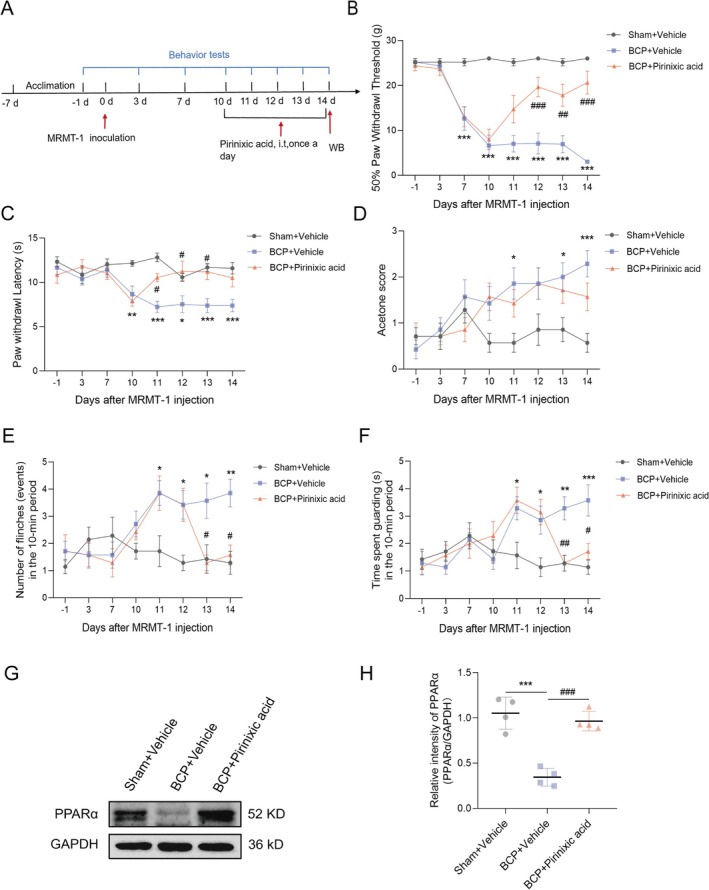
Analgesic effects of pirinixic acid in BCP model rats. (A) Experimental design involving the MRMT‐1 cell‐induced BCP model. (B–F) The rats were categorized into three groups: A Sham group receiving a placebo, a BCP group receiving a placebo, and a BCP group treated with pirinixic acid (i.t., 2 μg/10 μL). Behavioral assessments were conducted at predetermined time points to evaluate pain responses, including the 50% PWT (B), PWL (C), response to acetone (D), and spontaneous pain behaviors such as flinching (E) and guarding (F). These assessments were performed on Days −1, 3, 7, 10, 11, 12, 13, and 14. *n* = 7. Statistical analysis was performed via two‐way ANOVA to determine the significance of the results. Significant differences from the Sham + Vehicle group are denoted by *p < 0.05, **p < 0.01, and ***p < 0.001, whereas significant differences from the BCP + Vehicle group are denoted by ^#^ *p* < 0.05, ^##^ *p* < 0.01, and ^###^ *p* < 0.001. (G) Additionally, to examine the expression of spinal PPARα, Western blot analysis was carried out on the lumbar enlargement of the rats. (H) In comparison to vehicle treatment, pirinixic acid treatment significantly upregulated spinal PPARα expression. Analysis of the protein expression levels in four samples through statistical means disclosed a notable disparity (**p < 0.01) in comparison to the Sham + Vehicle group and a substantial difference (^###^ *p* < 0.001) in contrast to the BCP + Vehicle group—unpaired *t*‐test.

To ascertain the function of PPARα in the advancement of BCP, we administered the PPARα agonist pirinixic acid (Figure 5A). Behaviorally, this treatment significantly reduced the heightened mechanical sensitivity (Figure 5B), thermal sensitivity (Figure 5C), and spontaneous pain response (Figure 5E,F) caused by BCP. However, the acetone response was not significantly altered (Figure 5D). From postoperative Day 10 to Day 14, repeated daily injections of pirinixic acid significantly increased PPARαexpression levels in the treated group compared to the BCP group (Figure 5G,H). These findings underscore the critical role of PPARα in BCP.

### 3.5 Knockdown of PPARα in the Dorsal Horn of the Spinal Cord Induces Pain Behaviors in Normal Rats

**FIGURE 6:**
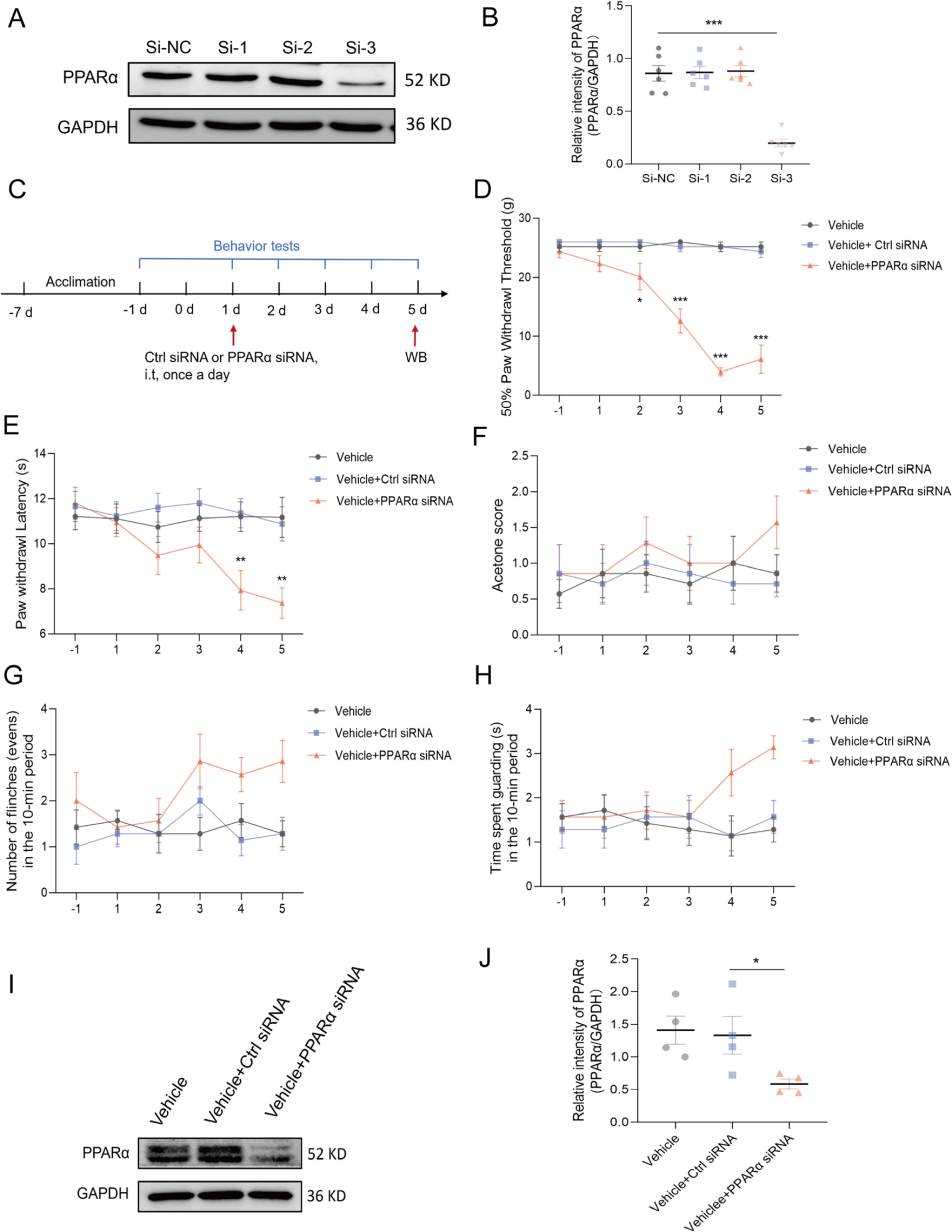
A reduction in PPARα expression in the dorsal horn neurons of the spinal cord induces pain behaviors in normal rats. (A) We noted a substantial reduction in the amount of PPARα protein in PC12 cells following exposure to various PPARα siRNAs (B), with siRNA3 exerting the most substantial effect (*n* = 6, ***p < 0.001 compared with the control siRNA group, analyzed by one‐way ANOVA). (C) For the in vivo experiments, the rats were divided into three groups: One received a vehicle, another was administered a combination of the vehicle and control siRNA, and the third received the vehicle supplemented with PPARα siRNA (2 μg/10 μL) (*n* = 7 for each group). Over a period from Days −1 to 5 (D–H), we conducted a series of behavioral tests to assess pain responses. The methods included measuring the 50% PWT (D) and PWL (E), evaluating the response to acetone (F), and observing spontaneous pain behaviors such as flinching (G) and guarding (H). The statistical significance of these behavioral assessments was determined via two‐way ANOVA, where the symbols *, **, and *** denote *p* values less than 0.05, 0.01, and 0.001, respectively, compared with the sham and vehicle groups. Additionally, (I) Western blot analysis was employed to evaluate spinal PPARα expression following PPARα siRNA injection. (J) The data indicated a considerable decline in spinal PPARα expression in the treatment group compared to the sham group. The statistical analysis of protein levels demonstrated a considerable variance among the four specimens (*n* = 4, *p < 0.05 in comparison with the vehicle plus control siRNA group, evaluated through unpaired *t*‐test).

To further explore the link between spinal PPARα expression and pain processing, we injected PPARα siRNA (Figure 6A,B) into normal rats via the epidural route (Figure 6C). The behavioral test results showed that siRNA injection led to the development of mechanical hyperalgesia (Figure 6D) and thermal hyperalgesia (Figure 6E). However, the response to acetone (Figure 6F) and spontaneous pain behavior (Figure 6G,H) were not significantly altered. The lack of significant changes in spontaneous pain and cold hyperalgesia may be attributed to the method of administration. Other routes of administration, such as intraperitoneal injection or oral gavage, should be considered for further study. Multiple epidural injections of PPARα siRNA once daily from postoperative Day 10 to Day 14 significantly decreased PPARα expression. As depicted in (Figure 6I,J), the expression of PPARα was notably reduced in the siRNA + Vehicle group in comparison to the Vehicle + Ctrl siRNA group. These results highlight the critical role of PPARα in modulating pain responses.

### 3.6 The Analgesic Effects of Anwulignan in a Rat Model of BCP Rely on PPARα

**FIGURE 7:**
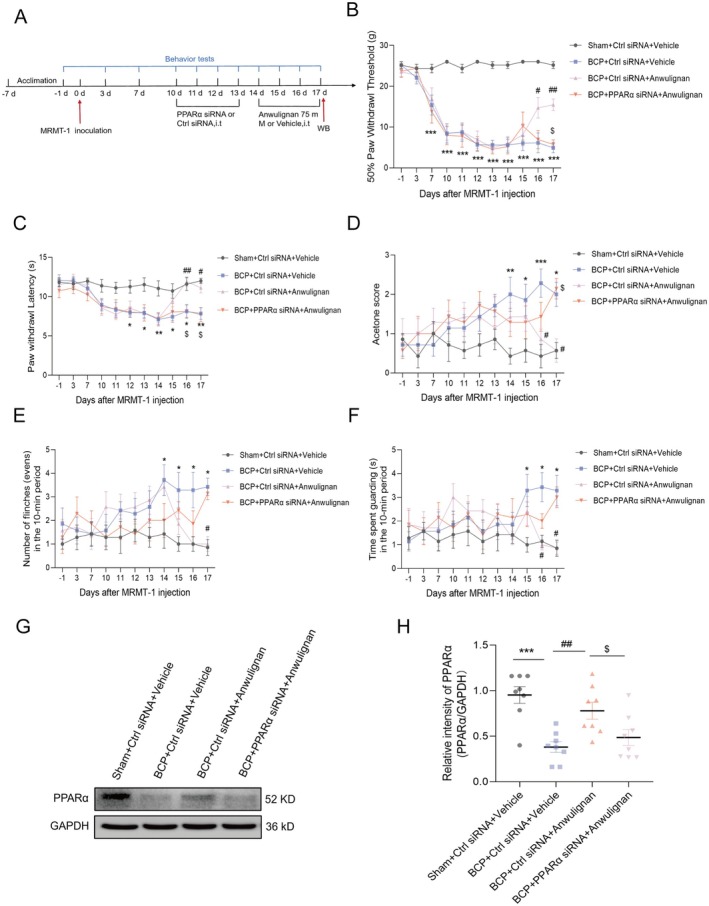
PPARα is essential for the analgesic effects of Anwulignan in BCP model rats. (A) A concise experimental timeline for the MRMT‐1 cell‐induced BCP model is presented. (B–F) Rats were randomly assigned to four groups (*n* = 7 per group): Sham + Ctrl siRNA + Vehicle, BCP + Ctrl siRNA + Vehicle, BCP + Ctrl siRNA + Anwulignan, and BCP + PPARα siRNA + Anwulignan. Pain‐related behavioral indicators, including 50% PWT (B), PWL (C), response to acetone (D), and spontaneous pain behaviors, such as flinching (E) and guarding (F), were assessed in the bone lesion–bearing rats at various time points from −1 to 17 days post‐treatment. Intrathecal injection of a fixed dose of PPARα siRNA (2 μg/10 μL) before Anwulignan injection negated the analgesic impact of Anwulignan (75 mM) on the affected hindlimbs of BCP model rats. Statistical significance is denoted by *p < 0.05, **p < 0.01, and ***p < 0.001 compared with the Sham + Ctrl siRNA + Vehicle group; ^#^ *p* < 0.05, ^##^ *p* < 0.01, and ^###^ *p* < 0.001 compared with the BCP + Ctrl siRNA + Vehicle group; and ^$^ *p* < 0.05 compared with the BCP + Ctrl siRNA + Anwulignan group, *n* = 7, according to two‐way ANOVA. (G, H) Illustrative protein expression data suggest that Anwulignan can abrogate the reduction in PPARα expression caused by BCP. Moreover, administering PPARα siRNA abrogated the increase in PPARα expression induced by Anwulignan in rats with BCP. ***p < 0.001 compared with the Sham + Ctrl siRNA + Vehicle group; ^##^ *p* < 0.01 compared with the BCP + Ctrl siRNA + Vehicle group; ^$^ *p* < 0.05 compared with the BCP + Ctrl siRNA + Anwulignan group, *n* = 8, according to unpaired *t* test.

To determine whether the pain‐relieving effects of Anwulignan on rats with BCP depend on PPARα, we administered siRNA (2 μg/10 μL) and Anwulignan (75 mM) intrathecally and observed any changes in pain‐related behaviors (Figure 7A). Assessments of mechanical allodynia (Figure 7B), thermal hyperalgesia (Figure 7C), and cold hyperalgesia (Figure 7D) revealed that the PPARα siRNA significantly counteracted the pain relief provided by Anwulignan; however, the data on spontaneous pain (Figure 7E,F) did not reveal significant inhibition of the analgesic effect of Anwulignan by PPARα. This discrepancy could be attributed to the limited sample size and the brief duration of the behavioral assessments using siRNA. Furthermore, our Western blot analysis confirmed that four consecutive days of intrathecal PPARα siRNA (2 μg/10 μL) treatment effectively blocked the increase in spinal PPARα expression. Anwulignan treatment significantly alleviates the levels of spinal PPARα in BCP rats (Figure 7G,H). Additionally, Anwulignan was found to alleviate BCP hypersensitivity by upregulating PPARα.

### 3.7 Analysis of Differential Gene Expression After the Knockdown of PPARα in PC12 Cells

**FIGURE 8:**
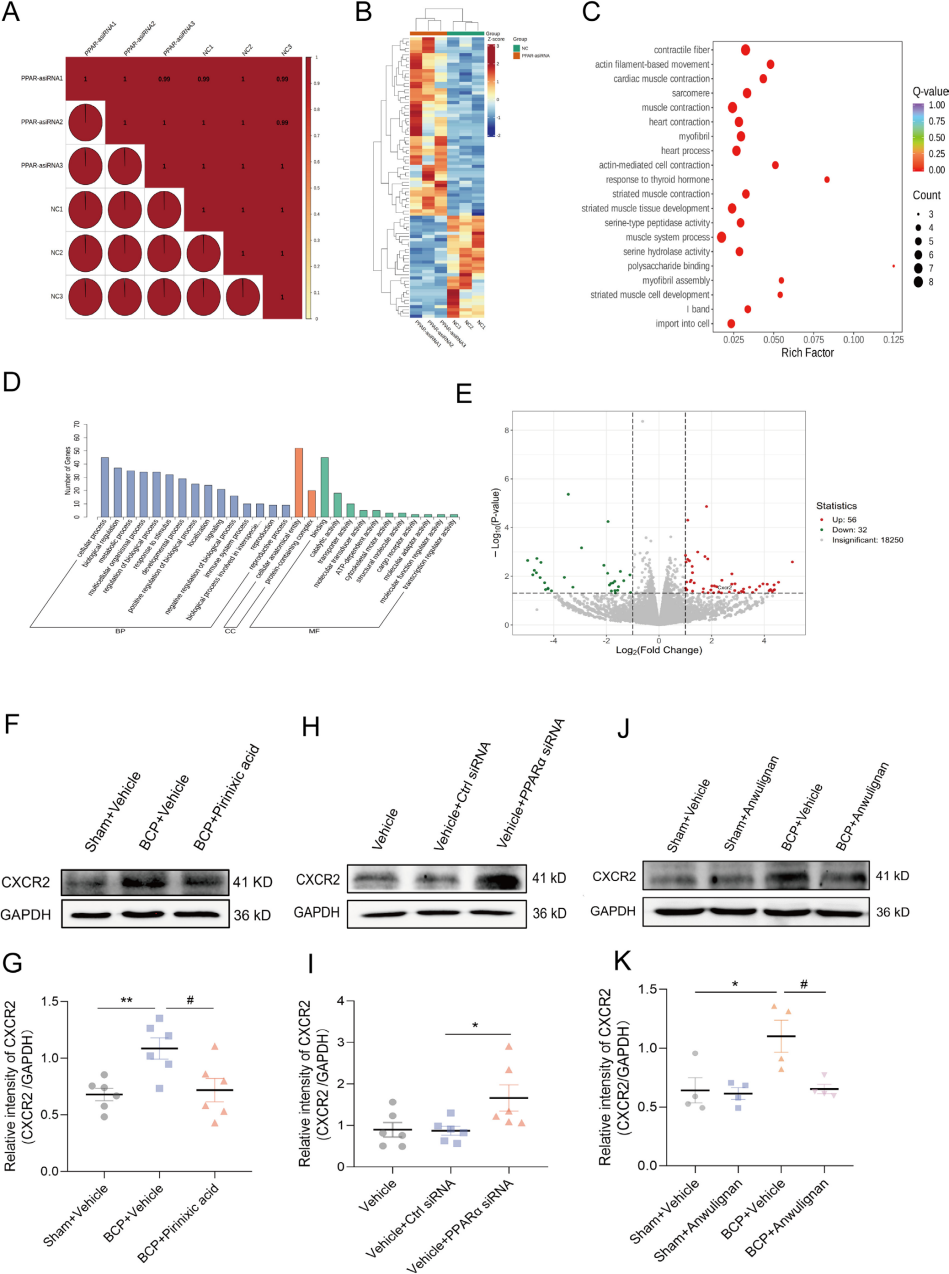
Comprehensive gene expression analysis to identify differences between the control group and the PPARα siRNA group. (A) Pearson's correlation coefficient was used to measure the correlation between biological replicates. (B) Gene expression patterns between the control and PPARα siRNA groups were differentiated using stratified clustering. (C) Pathway enrichment analysis using KEGG was used to identify pathways significantly enriched in the DEGs between the two groups. (D) Histograms were generated to classify the DEGs based on their enriched GO terms in biological process, molecular function, and cellular component. (E) Volcano plots visually represent the distribution of DEGs, distinguishing between upregulated and downregulated genes. (F, G) Immunoblotting analysis revealed that pirinixic acid, which functions as a PPARα agonist, significantly reversed the BCP‐induced increase in CXCR2 expression in the rat spinal cord (*p < 0.01 vs. the sham + vehicle group; ^#^ *p* < 0.05 vs. the BCP + vehicle group, unpaired *t*‐test). Additionally, (H, I) the downregulation of PPARα was associated with increased CXCR2 expression, as indicated by the immunoblotting results (*p < 0.05 vs. Vehicle + Ctrl siRNA group, unpaired *t*‐test). (J, K) Western blot analysis revealed that Anwulignan mitigated the BCP‐induced augmentation of CXCR2 expression in the spinal dorsal horn (*p < 0.01 vs. the sham + vehicle group; ^#^ *p* < 0.05 vs. the BCP + vehicle group, unpaired *t* test). These results suggest a regulatory interplay between PPARα and CXCR2 in BCP‐induced pain model rats, Pointing to a potential therapeutic target for the management of pain.

To normalize gene expression variations due to biological diversity, a standard methodology that involves the incorporation of multiple biological replicates into the experimental setup and then measuring the correlation between these replicates with Pearson's correlation coefficient (*r*) was used. The findings demonstrated a robust correlation among the replicate samples (Figure 8A). Moreover, the heatmap displayed distinct patterns in gene expression, highlighting significant differences (Figure 8B). To decipher the functions of the differentially expressed genes (DEGs), we executed KEGG and GO enrichment analyses on the DEGs that were identified between the two groups. The top 10 GO terms and 20 KEGG pathways were highlighted, with the KEGG analysis indicating a significant enrichment in the “complement and coagulation cascades” pathway among the DEGs (Figure 8C). The GO analysis revealed that the biological process category, the terms “metabolic process” and “immune system process” were the most enriched; in the cellular component (CC) category (Figure 8D), the terms “cellular, anatomical entity” and “protein‐containing complex” were the most enriched. In the molecular function (MF) category, the term “catalytic activity” was the most enriched. These results suggest that these genes and pathways are instrumental in modulating PPARα expression and pain processing. Following our RNA‐Seq analysis, which identified several DEGs in the transcriptome, we chose CXCR2 for additional validation to substantiate our findings. The Western blotting results provided further confirmation, which aligns with the RNA‐Seq data and attests to the accuracy of our observations. Our experiments revealed that the intrathecal administration of pirinixic acid in the dorsal horns of BCP model rats significantly abrogated the increase in CXCR2 expression induced by Anwulignan (Figure 8F,G). Furthermore, our study revealed that when PPARα was suppressed in the dorsal horns of normal rats, a notable increase in the protein expression of CXCR2 was detected. This finding, shown in Figure 8H,I, suggests a potential inverse relationship between PPARα levels and CXCR2 expression in the context of BCP. In addition, PPARα had a similar relationship with CXCR2 in the BCP model (Figure 8J,K). These results validate our RNA‐Seq data and highlight the potential therapeutic role of agonists such as pirinixic acid in altering the expression of genes linked to BCP, particularly those regulating CXCR2.

### 3.8 Relationship Between PPARα and CXCR2 Expression in the Spinal Dorsal Horn in BCP Model Rats

**FIGURE 9:**
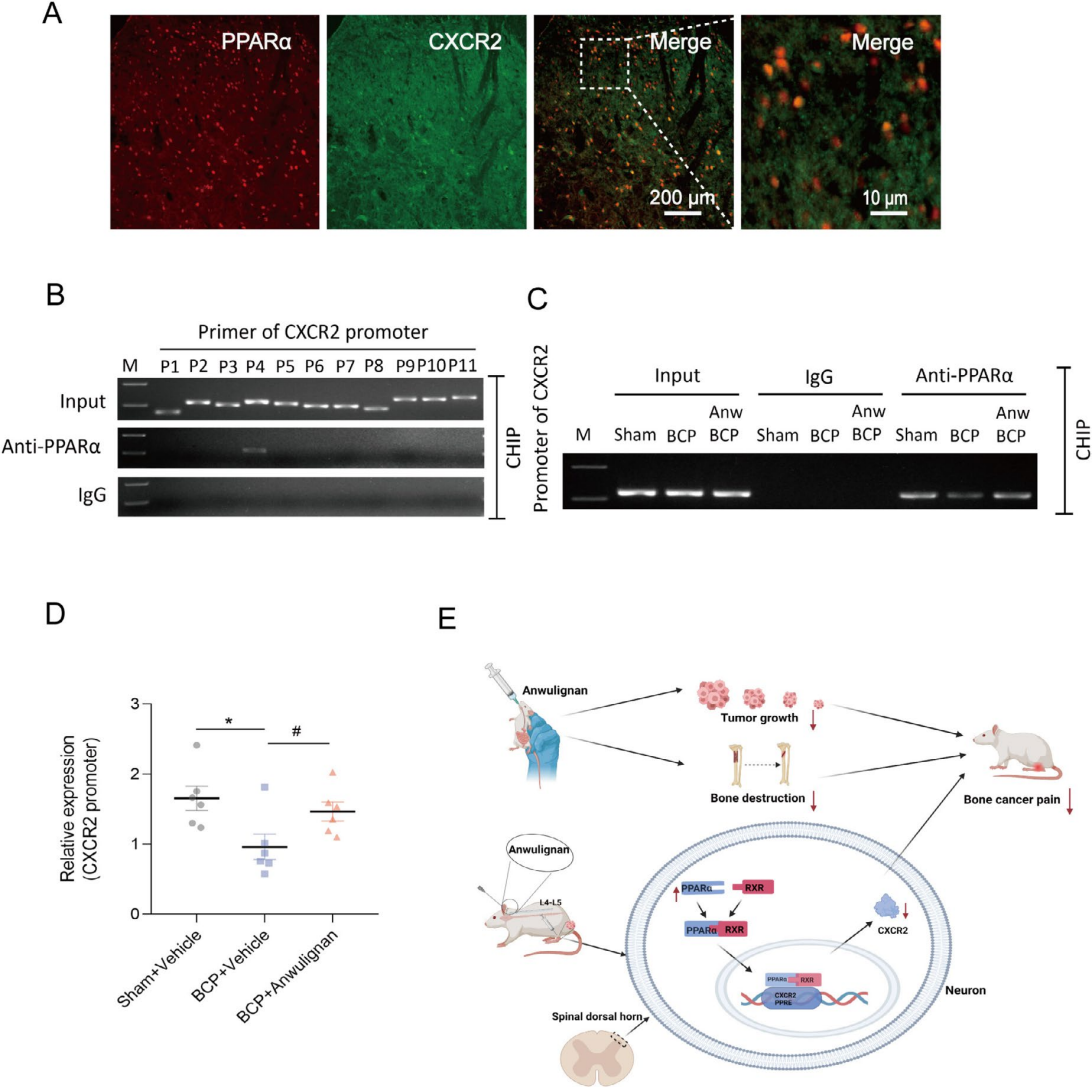
Relationship between PPARα and CXCR2 expression in the spinal dorsal horn in rats with BCP. (A) Immunofluorescence images showing the simultaneous localization of PPARα and CXCR2. (B) Representative nucleic acid gel images show that all primers designed for the CXCR2 promoter gene (P1 to P11) amplify the expected DNA fragments with the input DNA. (C) In ChIP experiments using anti‐PPARα, not IgG, only the expected product was detected with primer P4. (D) BCP significantly reduced the binding of PPARα to the CXCR2 promoter fragment in the dorsal horn of the spinal cord. However, multiple Anwulignan applications effectively abrogated this reduction (*p < 0.05 vs. the Sham + Vehicle group; ^#^ *p* < 0.05 vs. the BCP + Vehicle group; unpaired *t*‐test). *n* = 6. (E) Anwulignan enhances the expression of PPARα in the spinal dorsal horn of rats, facilitating its binding to RXR and forming an active PPARα/RXR complex. This complex translocates to the nucleus, inhibits CXCR2 expression, and alleviates bone cancer pain (BCP) while reducing tumor‐induced bone destruction and burden.

CXCR2 levels were found to be closely associated with PPARα expression, which required further validation. The immunofluorescence results revealed that PPARα colocalized with CXCR2 (Figure 9A). We also used ChIP to assess the effects of BCP‐induced PPARα knockdown on the binding of PPARα to the CXCR2 promoter. The results revealed that all primers targeting the CXCR2 promoter region (P 1 to P 11) produced the expected product when the amplification reaction was performed on DNA fragments treated without an anti‐PPARα antibody (Figure 9B). However, the expected product of primer P4 was detected only when the DNA fragment was treated with an anti‐PPARα antibody and not with IgG. Thus, primer P4 was used in the subsequent agarose gel electrophoresis experiments (Figure 9C). The results revealed that the intensity of the CXCR2 promoter fragment was similar in all the input DNA samples. BCP significantly reduced the amount of the CXCR2 promoter fragment that was precipitated by the anti‐PPARα antibody. In comparison to the samples from the sham group, the samples in the BCP group demonstrated a marked decrease in the levels of the CXCR2 promoter fragment, but no changes in the precipitation of IgG were detected. Anwulignan abrogated this reduction (Figure 9D). The greyscale value analysis also revealed that the anti‐PPARα antibody participated in CXCR2 promoter DNA fragmentation. The fragmentation of the CXCR2 promoter DNA in the BCP group was significantly decreased. In contrast, an augmentation was witnessed in the BCP + Anwulignan group. These results imply that Anwulignan counteracts the BCP‐induced diminution in the binding of PPARα to the CXCR2 promoter.

## 4 Discussion

BCP is a severe issue that affects individuals of all ages and has clinical, economic, and social implications. Despite the existence of several treatments for BCP, approximately 45% of patients continue to experience insufficiently managed pain and require further treatment [34]. Emerging preclinical evidence indicates that Anwulignan, which possesses anti‐inflammatory and antitumor properties, has therapeutic potential for treating BCP [8, 35]. Our findings indicate that Anwulignan alleviates pain through the PPARα/CXCR2 signaling pathway. The combined function of Anwulignan in reducing the tumor burden and related bone destruction may account for the robust analgesic effects observed in our in vivo investigation.

Anwulignan is a lignan that is extracted from the seeds of *M. fragrans* . Anwulignan serves as a safeguard for skin keratinocytes against UVB radiation‐induced harm and curbs the expression of matrix metalloproteinase‐9 (MMP‐9) and cyclooxygenase‐2 (COX‐2) by reducing the activation of mitogen‐activated protein kinases (MAPKs) and the phosphatidylinositol‐3‐kinase/protein kinase B (PI3K/Akt) pathway [36]. Moreover, Anwulignan protects dopaminergic neurons [37] Nevertheless, the involvement of Anwulignan in pain control remains unexplored. Previous research has shown that intrathecal injection facilitates the distribution of the drug to the spinal cord and DRG [38]. Therefore, to explore the analgesic effect of Anwulignan on BCP, we administered the drug intrathecally continuously from 10 to 14 days after inoculating MRMT‐1 cells. Using gabapentin as a positive control, when administered in more significant amounts, Anwulignan has analgesic properties comparable to those of gabapentin, a frequently prescribed medical intervention for the management of pain [39, 40, 41]. These findings suggest that Anwulignan has the potential to be effective in clinical applications.

After confirming the analgesic effect of Anwulignan, we further explored the underlying mechanisms of its pain‐relieving action. Previous research has suggested that PPARα is a critical molecular target of Anwulignan [14]. We employed molecular docking and cellular thermal shift assays to substantiate the Anwulignan and PPARα relationship. In the rat model of neuropathic pain induced by spinal nerve ligation, increased PPARα protein levels were observed in the spinal cord on the injured side [13]. Moreover, a reduction in spinal PPARα protein levels was linked to increased peripheral inflammation and heightened inflammatory pain sensitivity in obese rats with diet‐induced [42]. These results imply that PPARα holds a crucial position in the process of pain. However, the specific role of PPARα in BCP in rats has not yet been explored. Considering that the spinal dorsal horn is the leading integrator of peripheral sensory input and its effect on pain perception (due to the excitability of spinal neurons) [43, 44], we experimentally increased and reduced PPARα levels in the spinal cords of BCP model rats and healthy rats. Our findings suggest a close association between PPARα and BCP.

To explore downstream molecules related to PPARα, we conducted whole‐transcriptome sequencing and found that CXCR2 expression was greatly affected by PPARα silencing. Previous research has demonstrated that inhibiting CXCL6 can upregulate PPARα expression, whereas the PPARα inhibitor GW6471 can partially counteract this effect [45]. Moreover, PPARγ activates the CXCR2 promoter by binding to the PPAR response element (PPRE) [22]. Therefore, based on the transcriptomic sequencing results, we propose a potential link between PPARα and the chemokine receptor family CXCR2. Concurrently, Related investigations demonstrate that CXCR2 holds a crucial role in BCP, and the suppression of CXCR2 by means of the selective antagonist SB225002 alleviated BCP [19]. Moreover, previous research has demonstrated that CXCR2 is involved in promoting bone colonization and the metastasis of breast cancer tumor cells, closely linking it to cancer progression [46]. Meanwhile, Liquiritin notably mitigates BCP in rats by inhibiting the activation of the CXCL1 –CXCR2 signaling pathway between spinal astrocytes and neurons [47]. These findings further demonstrate the close association between CXCR2 and bone cancer pain.

In recent years, natural products have emerged as a prominent area of research in oncology due to their affordability, long‐standing existence, and multitarget effects [48, 49]. Studies have shown that Anwulignan can induce apoptosis in human promyelocytic leukemia (HL‐60) cells by activating caspase‐3 [50]. Additionally, Anwulignan has been found to inhibit the growth of non‐small cell lung cancer [7] and the metastasis of colorectal cancer [8]. These findings suggest that Anwulignan could serve as a potential therapeutic agent for reducing tumor burden in rats with BCP. In vivo and in vitro experiments have shown that Anwulignan can strikingly suppress tumor growth(Figure S3). Furthermore, our findings indicate that Anwulignan mitigates bone destruction induced by bone cancer(Figure S2). Previous studies have demonstrated that Anwulignan can promote osteoblast differentiation in vitro [10]. Given that the overactivation of osteoclasts or reduced osteoblast activity is closely associated with bone destruction, immunohistochemical staining revealed that Anwulignan significantly increased the stained area corresponding to osteoblasts, with no notable changes in osteoclast staining(Figure S2D,E). These findings are consistent with previous research, suggesting that Anwulignan stimulates osteoblast differentiation, exerting an anabolic effect on bone metabolism and attenuating bone destruction.

While Anwulignan demonstrates notable analgesic effects, prolonged or high‐dose use could pose risks of toxicity to vital organs like the liver and kidneys. Additionally, interactions with other treatments, such as chemotherapy or opioids, may elevate the risk of adverse reactions. Potential immune modulation and CNS effects, like sedation, could also occur. Therefore, a thorough evaluation of its safety profile, including long‐term toxicity and drug interactions, is essential for assessing its clinical applicability in bone cancer pain management.

## 5 Conclusion

Our findings suggest that Anwulignan mitigates BCP through a distinct synergistic mechanism, which involves the upregulation of PPARα expression to inhibit the expression of CXCR2, a reduction in tumor burden, and the promotion of osteoblast synthesis.

## Author Contributions

**Yueliang Wang:** conceptualization, formal analysis, investigation, writing – original draft, writing – review and editing. **Qingying Liu:** investigation, formal analysis, writing – review draft. **Yingying Jiang:** investigation. **Longfei Mao:** resources. **Mohamed Zoubaa:** resources. **Jian Wang:** resources, funding acquisition, writing – review and editing. **Huilian Bu:** resources, funding acquisition, writing – review and editing. **Minyu Ma:** writing – review and editing. **Jingjing Yuan:** writing – review and editing. **Jing Cao:** writing – review and editing. **Xiaochong Fan:** conceptualization, supervision, funding acquisition, project administration, visualization, writing – review and editing.

## Conflicts of Interest

The authors declare no conflicts of interest.

## Supporting information


Appendix S1.

Figure S1.

Figure S2.

Figure S3.

## Data Availability

Research data are not shared.
