# Supplementary material for: Anwulignan Alleviates Bone Cancer Pain by Modulating the PPARα/CXCR2 Signaling Pathway in the Rat Spinal Cord
Source: CNS Neurosci Ther. 2025 Mar 13;31(3):e70302. doi: 10.1111/cns.70302 (PMC11904945; doi:10.1111/cns.70302)
Supplement: Supplementary file 1 — Appendix S1. [file CNS-31-e70302-s003.docx]

**Materials and methods**

1. Rat tibiae were histologically evaluated and stained for tartrate-resistant acid phosphatase (TRAP) and alkaline phosphatase (ALP).

The tibias were removed and placed in the fixative at 4°C for 48 hours. The bone was demineralized and kept in a 10% EDTA solution for ten days. Just before sectioning, the tibia samples were dehydrated through a gradient of increasing ethanol concentrations, from 30% to 100%, before being embedded in paraffin. Sections of trabecular bone, 5 micrometers thick, were prepared from the tibia. These sections were stained with TRAP or ALP [1]. Static histomorphometric analysis of the bone was performed to determine the percentage of the area occupied by osteoclasts and osteoblasts in the tissue sections.

2. Colony formation assay

The MRMT-1 cells were carefully treated to fulfill the experiment's needs. After the cells adhered the cells were incubated for 48 hours. Upon the cells being rinsed thrice with PBS for the purpose of eliminating any residual medium, the culture process was completed by fixing the cells with a 4% paraformaldehyde solution for 20 minutes at room temperature to maintain the cell shape. The cells were stained by immersing them in a 0.1% crystal violet solution for 15 minutes at ambient temperature. Next, the cells were rinsed with PBS to remove any remaining dye, allowed to air dry, photographed, and analyzed.

3. CCK-8 assay

The cells were placed in the wells of a 96-well plate in accordance with the experimental configuration. Following preincubation, various concentrations of Anwulignan were introduced into the wells. To quantify the viability of the cells, ten microlitres of CCK-8 reagent were meticulously added to each well for 1 to 4 hours to allow the reaction. Absorbance was precisely measured at a wavelength of 450 nm.

4. In vivo imaging

Cy5.5 is a fluorescent probe in the near-infrared spectrum to study their bodily distribution via immunofluorescence. The solution was administered intravenously to the rats at 0.2 milligrams per kilogram of body weight [2,3]. The biodistribution was then observed with the IVIS Spectrum imaging system.

**Results**

1. **Anwulignan prevents cancer-induced bone destruction**

Bone destruction often occurs as a result of bone cancer, which can cause BCP. In our model, there was a noticeable increase in bone destruction in the proximal region of the tibia, while the distal region was largely unaffected. The difference was not statistically significant (Supplementary Fig. 2 A and B). However, rats treated with Anwulignan exhibited lower bone destruction scores on day 21 following MRMT-1 cell injection than those of BCP model rats.

To delve deeper into the bone microarchitecture, we utilized micro-CT to perform an ex vivo three-dimensional reconstruction analysis of the proximal tibia, which harbored the tumor. On day 21 after BCP induction, the loss of trabecular bone loss and the number of cortical bone lesions were decreased in the Anwulignan-treated group compared to the BCP+Vehicle group (Supplementary Fig. 2 C).

Previous investigations have revealed a complex relationship between cancer-induced bone destruction and the roles of osteoclasts and osteoblast. Moreover, there is evidence that cancer cells may aggravate BCP by promoting osteoclastogenesis and that newly formed osteoclasts release enhanced bone resorption, leading to bone deterioration and painful fractures. Thus, cancer cells in the bone tumor microenvironment can induce bone pain through both direct and indirect pathways. Given that Anwulignan can protect against bone destruction, our goal was to determine whether osteoclast and osteoblast development were involved in this protective effect. To investigate this question thoroughly, we measured the area occupied by osteoclasts and osteoblasts proximal to the tibia of rats injected with MRMT-1 cells or treated with a vehicle on day 21 after inoculation. Our experimental findings revealed that Anwulignan did not significantly alter the region characterized by osteoclasts. On the other hand, we noticed a significant increase in the osteoblast region (Supplementary Fig. 2 D and E). This result is consistent with previous studies that suggest that Anwulignan may exert its bone-preserving effects by stimulating osteoblast differentiation, thereby potentially reducing the incidence of bone destruction. In clinical practice, a critical comorbidity for patients with advanced cancer and bone metastasis is a reduction in or loss of mobility, leading to functional impairment and decreased quality of life. We evaluated locomotor activity via the open field test to determine whether Anwulignan can improve motor function in rats with BCP. The results indicated that Anwulignan treatment did not enhance motor function in BCP model rats (Supplementary Fig.1).

1. **Anwulignan suppresses the bone tumor burden**

To determine the influence of Anwulignan on both the colony formation and proliferation of MRMT-1 cells, as shown below. Cell growth was assessed via crystal violet staining (Supplementary Fig. 3 A and B) and a CCK-8 assay (Supplementary Fig. 3 C and D). The results demonstrated that Anwulignan inhibited the ability of MRMT-1 cells to form colonies and proliferate in a concentration-dependent manner.

Studies have shown that the in vivo biodistribution and accumulation of Cy5.5 were monitored via an optical microscope that detects fluorescence signals. There needs to be more information regarding the distribution and accumulation of free Cy5.5 dye in mouse tissues. Study showed that the fluorescence intensity in various organs decreased from 0.5 to 24 hours after intravenous injection, peaking at approximately 4 hours after injection. Therefore, the fluorescence signals at the tumor site is evaluated approximately 4 hours after injection. The results revealed a significant decrease in tumor fluorescence intensity at the injection site after one week of continuous gavage administration (vehicle or Anwulignan, 40 mg/kg i.g.) on day 21 following MRMT-1 cell injection (Supplementary Fig. 3 E and F).

Osteolytic bone destruction caused by cancer often leads to fractures and is associated with decreased survival rates. On day 21, after the MRMT-1 cell injection, the rats were euthanized, and the tumor-bearing tibias were removed. The results revealed that Anwulignan significantly suppressed local tumor growth (Supplementary Fig. 3 G).

**Reference**

[1] Wang K, Gu Y, Liao Y, et al. PD-1 blockade inhibits osteoclast formation and murine bone cancer pain. J Clin Invest. 2020;130(7):3603-3620.

[2] Fu Y, He G, Liu Z, et al. DNA Base Pairing-Inspired Supramolecular Nanodrug Camouflaged by Cancer-Cell Membrane for Osteosarcoma Treatment. Small. 2022;18(30): e2202337.

[3] Hue JJ, Lee HJ, Jon S, et al. Distribution and accumulation of Cy5.5-labeled thermally cross-linked superparamagnetic iron oxide nanoparticles in the tissues of ICR mice. J Vet Sci. 2013;14(4):473-479.


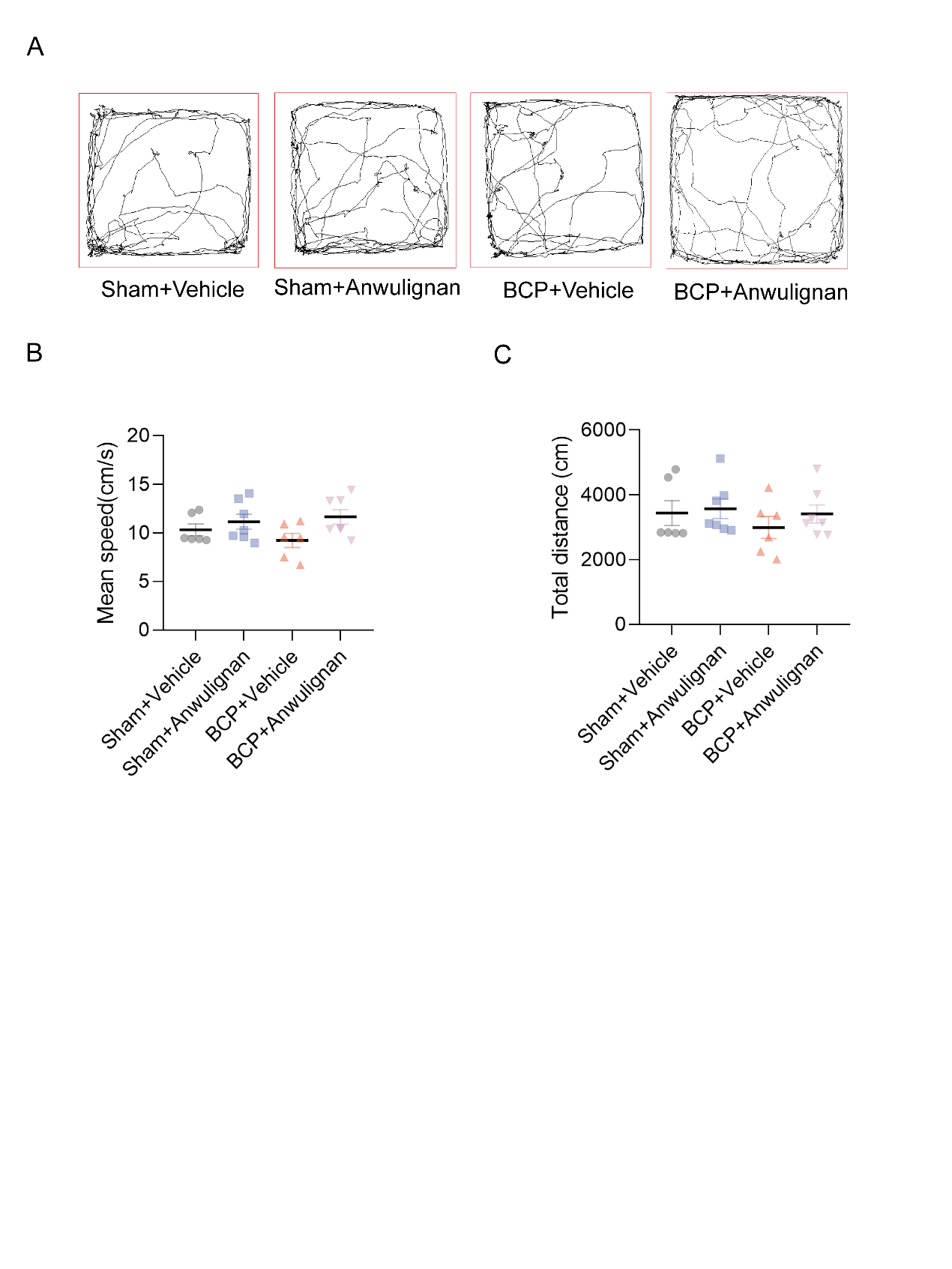


**Supplementary Fig. 1.** The impact of Anwulignan on locomotor function in rats with BCP. (A) Horizontal movement traces in the OFT of the Sham+Vehicle group, Sham+Anwulignan group, BCP+Vehicle group, and BCP+Anwulignan group. (B, C) The OFT result showed that there was no significant difference in the total distance and average speed between the Sham+Vehicle group, Sham+Anwulignan group, BCP+Vehicle group, and BCP+Anwulignan group (p>0.05)


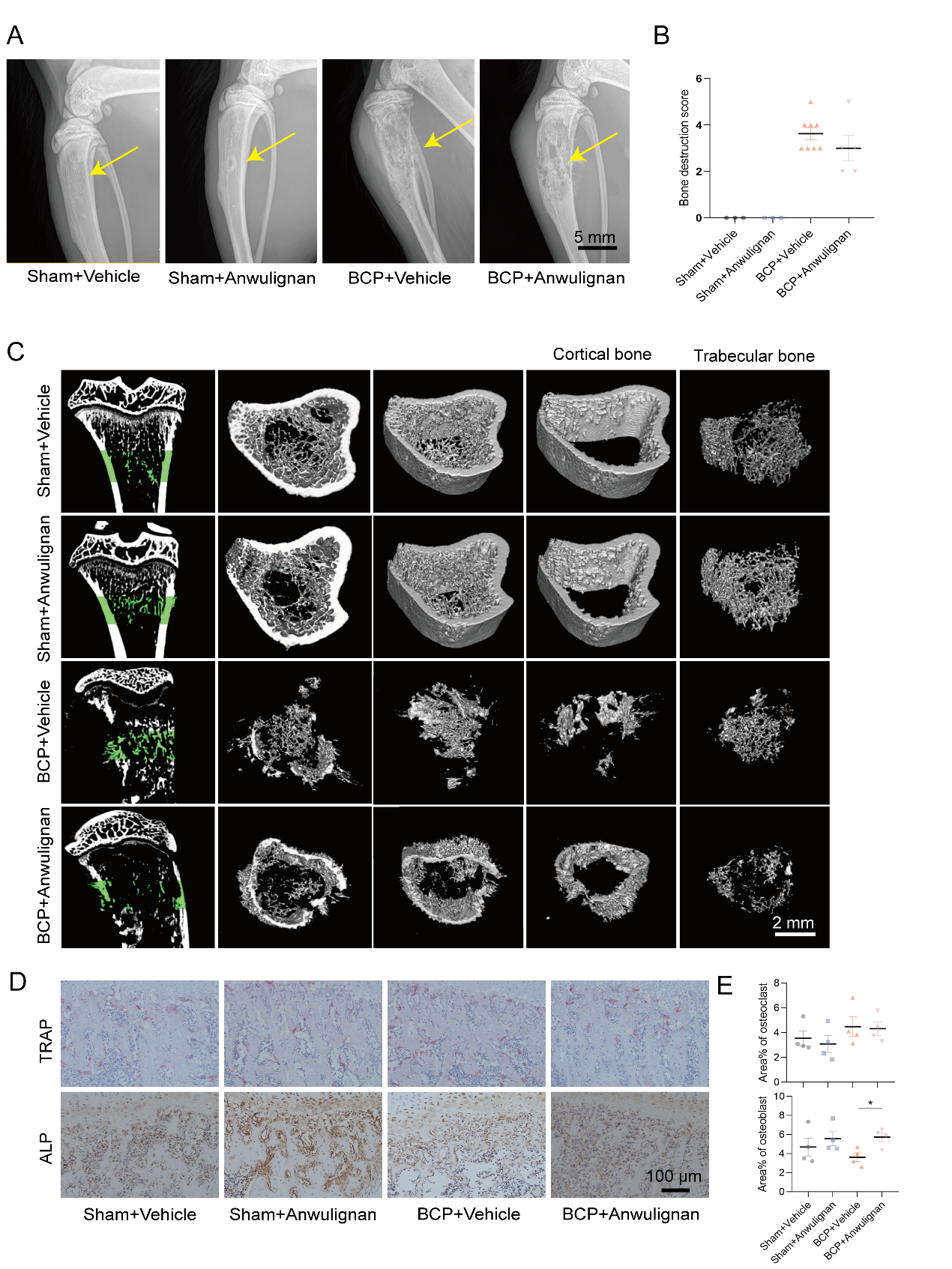


**Supplementary Fig. 2.** Anwulignan attenuates cancer-induced bone destruction. (A) Representative X-ray images of tibias from rats treated with either vehicle or Anwulignan (40mg/kg i.g.) for one week via continuous gavage on day 21 post-MRMT-1 cell injection in the Sham+Vehicle, Sham+Anwulignan, BCP+Vehicle, and BCP+Anwulignan groups are shown, with a scale reference of 5 mm. The arrows indicate areas of bone lesions. (B) Quantitative analysis of X-ray data. n=3-8; unpaired t test. (C) Micro-CT images captured on day 21 post-MRMT-1 cell injection illustrate the deterioration of trabecular and cortical bone in the proximal tibia of tumor-bearing rats, with a scale bar of 2 mm. (D) Representative images of TRAP and ALP staining (left) showing osteoclasts and osteoblasts in the proximal tibiae of tumor-bearing rats, with a scale bar of 100 µm. (E) Quantitative analysis of the staining, n=4. Statistical significance (*p < 0.05) compared with the BCP+Vehicle group was determined via unpaired t-test.


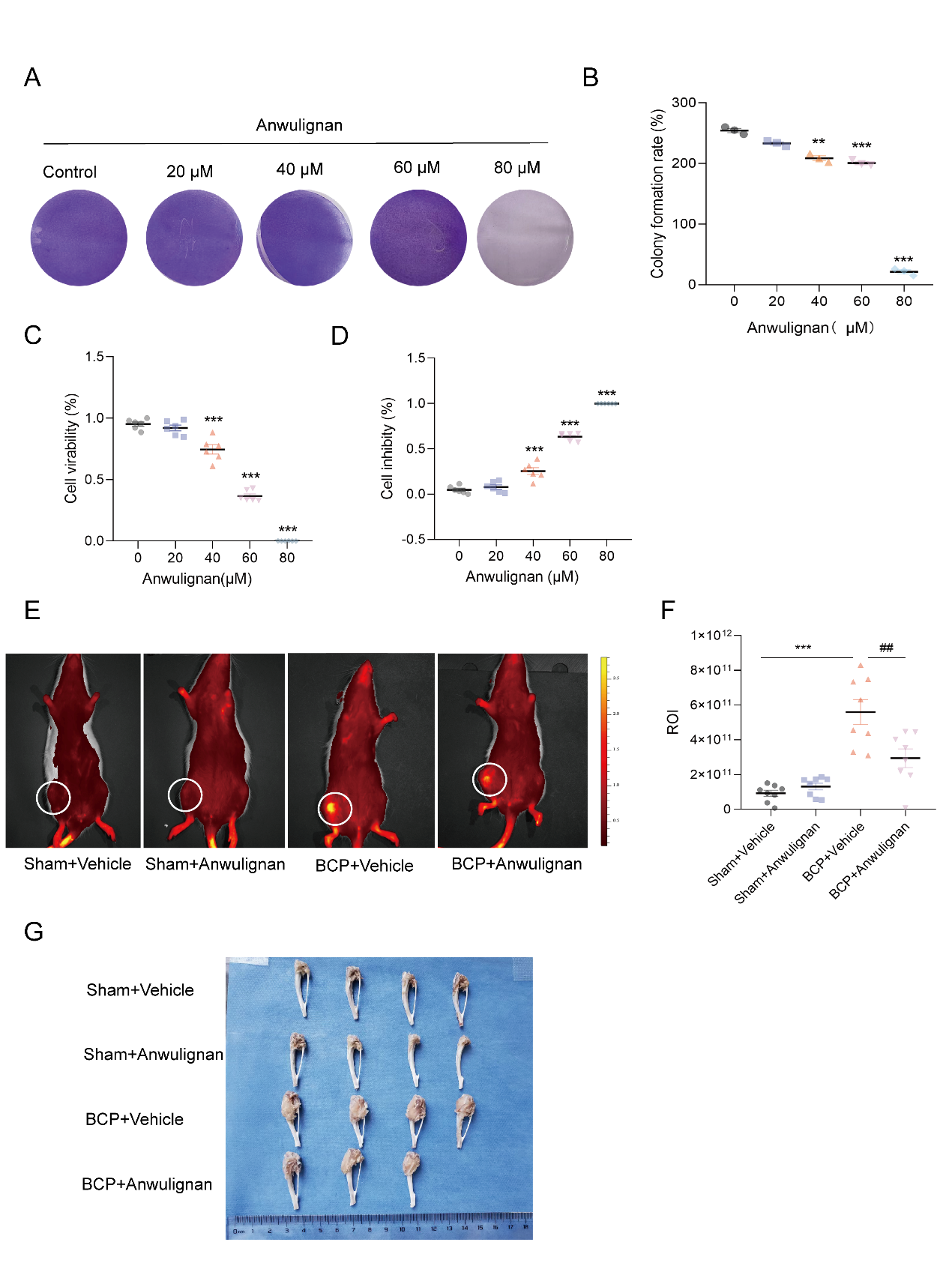


**Supplementary Fig. 3.** Anwulignan reduces bone tumor volume. (A) Light microscopy images showing the colony formation of MRMT-1 cells after treatment with different concentrations of Anwulignan (ranging from 0 to 80 μM). (B) Numerical data for this experiment are presented with three replicates per condition. Significant differences were marked with * * P <0.01 and * * * p <0.001 via one-way ANOVA. (C, D) The effect of Anwulignan on MRMT-1 cell growth was assessed via a CCK-8 assay, which was used to assess cell viability and cell inhibity. A significant reduction in cell proliferation was observed in response to the Anwulignan treatment, with six replicates per condition. Differences were statistically significant compared with the controls via one-way ANOVA, and the results are labeled * * * p <0.001. (E) In vivo images of bone tumors from rats are shown after one week of continuous gavage administration (vehicle or Anwulignan, 40 mg/kg i.g.) on day 21 after MRMT-1 cell injection. (F) Quantitative analysis of these images, with eight replicates per condition. Significant differences are indicated by * * *P <0.001 compared with the Sham + Vehicle group and significant differences from the BCP + Vehicle group by # # P <0.01 via unpaired t test. (G) In vitro images of bone tumors from rats treated with vehicle or Anwulignan on day 14 after cell injection are provided.
