# Supplementary figures and images for: Anwulignan Alleviates Bone Cancer Pain by Modulating the PPARα/CXCR2 Signaling Pathway in the Rat Spinal Cord
Source: CNS Neurosci Ther. 2025 Mar 13;31(3):e70302. doi: 10.1111/cns.70302 (PMC11904945; doi:10.1111/cns.70302)

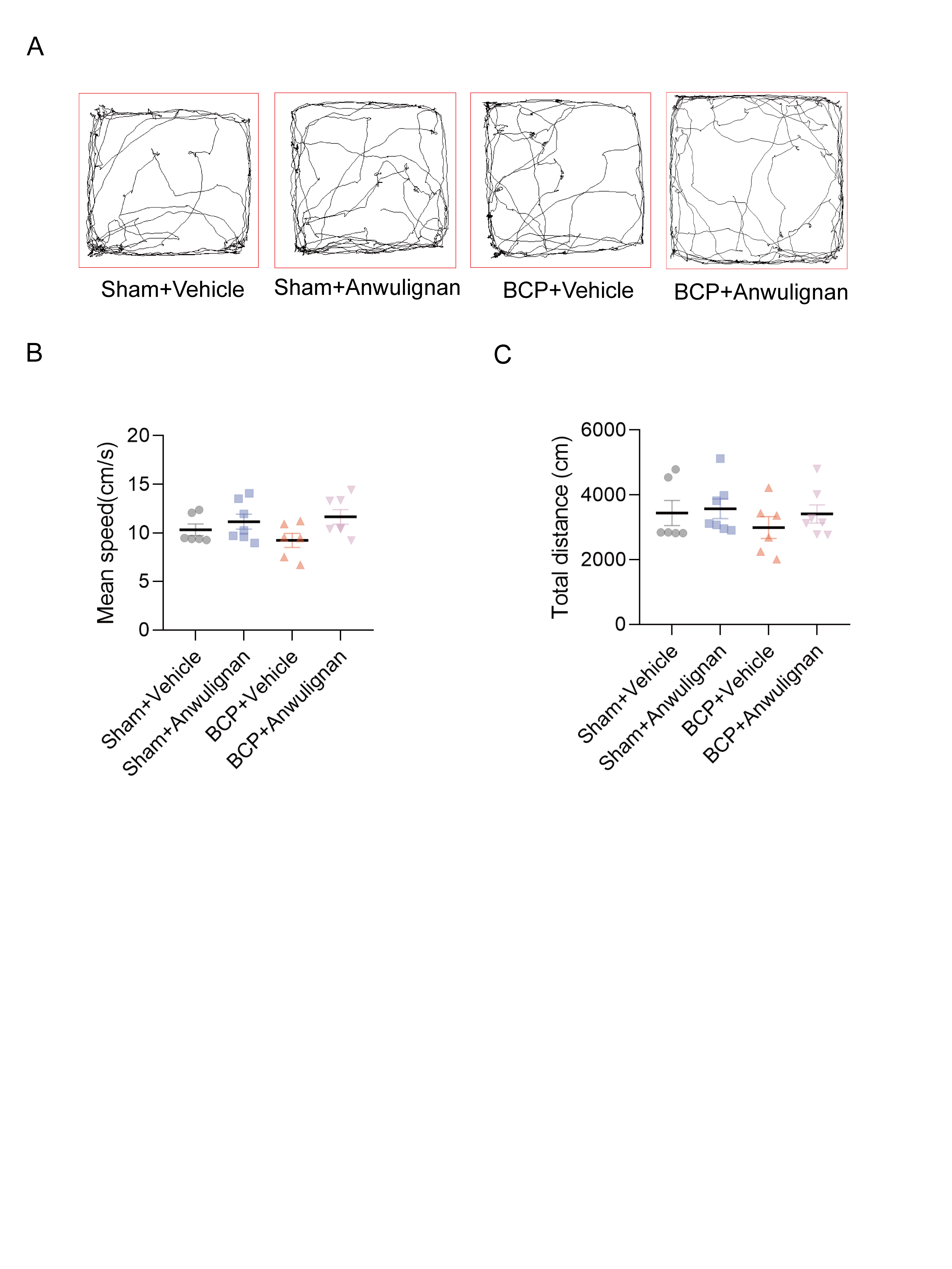

Supplement: Supplementary file 2 — Figure S1. [file CNS-31-e70302-s002.tif]

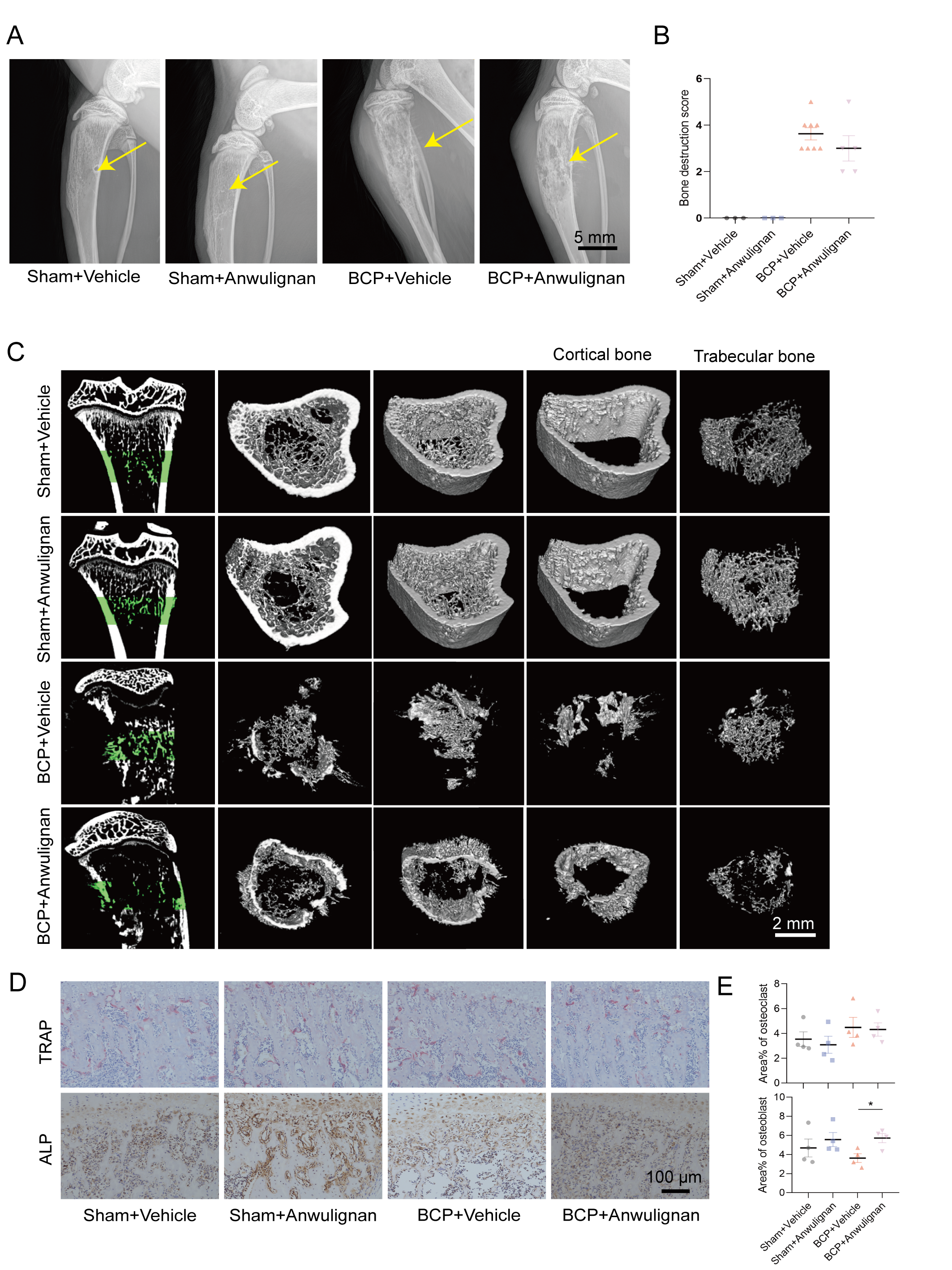

Supplement: Supplementary file 3 — Figure S2. [file CNS-31-e70302-s001.tif]

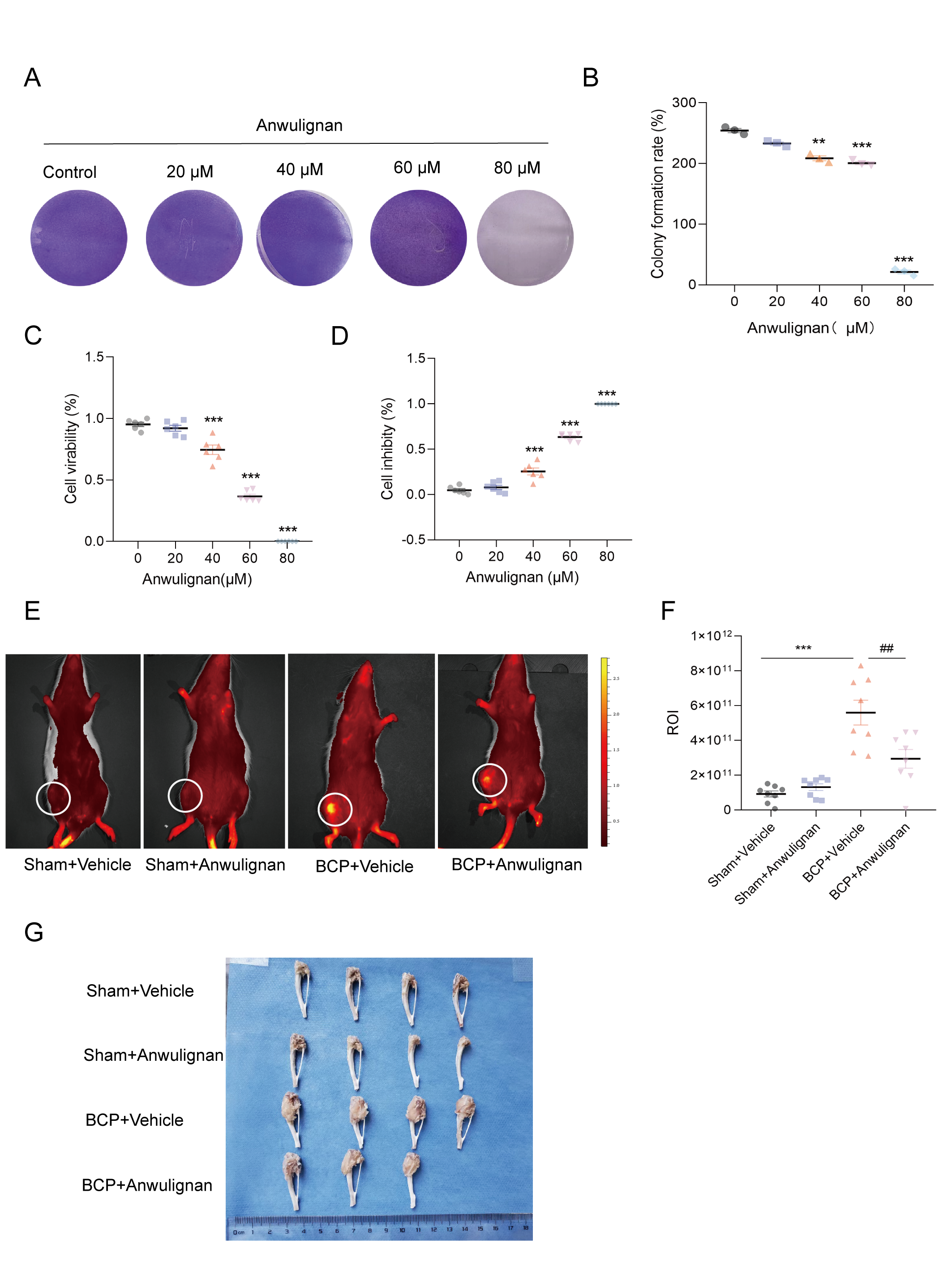

Supplement: Supplementary file 4 — Figure S3. [file CNS-31-e70302-s004.tif]
